# Identification of *nosZ*-expressing microorganisms consuming trace N_2_O in microaerobic chemostat consortia dominated by an uncultured *Burkholderiales*

**DOI:** 10.1038/s41396-022-01260-5

**Published:** 2022-06-08

**Authors:** Daehyun D. Kim, Heejoo Han, Taeho Yun, Min Joon Song, Akihiko Terada, Michele Laureni, Sukhwan Yoon

**Affiliations:** 1 Department of Civil and Environmental Engineering, Korea Advanced Institute of Science and Technology (KAIST), Daejeon, 34141 Korea; 2 Department of Chemical Engineering, Tokyo University of Agriculture and Technology, Tokyo, 184-8588 Japan; 3 Department of Biotechnology, Delft University of Technology, Delft, The Netherlands; 4 Department of Water Management, Delft University of Technology, Delft, The Netherlands

**Keywords:** Microbial ecology, Water microbiology, Climate-change ecology, Functional genomics, Next-generation sequencing

## Abstract

Microorganisms possessing N_2_O reductases (NosZ) are the only known environmental sink of N_2_O. While oxygen inhibition of NosZ activity is widely known, environments where N_2_O reduction occurs are often not devoid of O_2_. However, little is known regarding N_2_O reduction in microoxic systems. Here, 1.6-L chemostat cultures inoculated with activated sludge samples were sustained for ca. 100 days with low concentration (<2 ppmv) and feed rate (<1.44 µmoles h^−1^) of N_2_O, and the resulting microbial consortia were analyzed via quantitative PCR (qPCR) and metagenomic/metatranscriptomic analyses. Unintended but quantified intrusion of O_2_ sustained dissolved oxygen concentration above 4 µM; however, complete N_2_O reduction of influent N_2_O persisted throughout incubation. Metagenomic investigations indicated that the microbiomes were dominated by an uncultured taxon affiliated to *Burkholderiales*, and, along with the qPCR results, suggested coexistence of clade I and II N_2_O reducers. Contrastingly, metatranscriptomic *nosZ* pools were dominated by the *Dechloromonas*-like *nosZ* subclade, suggesting the importance of the microorganisms possessing this *nosZ* subclade in reduction of trace N_2_O. Further, co-expression of *nosZ* and *ccoNO*/*cydAB* genes found in the metagenome-assembled genomes representing these putative N_2_O-reducers implies a survival strategy to maximize utilization of scarcely available electron acceptors in microoxic environmental niches.

## Introduction

Climate change, brought about by a dramatic increase in greenhouse gas (GHG) emissions over the last two centuries, is one of today’s most serious environmental concerns [1, 2]. Nitrous oxide (N_2_O) is one of the three major GHGs along with carbon dioxide (CO_2_) and methane (CH_4_) [1, 3]. The concentration of N_2_O in the atmosphere has increased steadily over the past century due primarily to the increased anthropogenic nitrogen inputs to the environment and currently stands at 332 ppb, a concentration approximately 20% higher than in the pre-industrial era [3]. As N_2_O has 298 times higher per-mole global warming potential than CO_2_ over a 100-year scale, the contribution of N_2_O to the net global warming potential is substantial (estimated to be 6.2%) despite its low atmospheric concentration [2]. Thus, scientific and engineering endeavors to understand and mitigate N_2_O emissions are an indispensable part of global efforts to curb climate change and ensure a sustainable future.

Unlike CO_2_, > 90% of N_2_O emitted to the atmosphere has a biological origin [3]. Net emission from an environmental N_2_O source is determined by the difference between the rates of its production and removal. While a multitude of microbial-mediated processes including nitrification and denitrification result in production of N_2_O, N_2_O can be removed only via biological reduction catalyzed by a family of enzymes referred to as nitrous oxide reductases (NosZ) [4, 5]. This reaction had long been regarded merely as a part of the chain of reactions constituting the denitrification pathway, i.e., the stepwise reduction of NO_3_^−^ to N_2_ via NO_2_^−^, NO, and N_2_O; however, the significance of the reaction as a growth-supporting standalone terminal electron acceptor reaction has only recently been recognized, as genomic and physiological investigations have repeatedly suggested that denitrification occurs largely in a modular manner in the environment and also that a substantial portion of *nosZ*-possessing microorganisms lack either *nir* gene [6–9]. Further, discovery of a novel *nosZ* clade, later termed clade II *nosZ*, led to recognition of the true diversity of *nosZ* and *nosZ*-harboring microorganisms [6, 10]. These recent findings altogether signified the potential importance of *nosZ*-possessing N_2_O-reducing microorganisms as a N_2_O sink.

The *nosZ*-harboring N_2_O reducers are often categorized into two subgroups based on the type of *nosZ* they possess [7, 11]. The clade I and clade II NosZ have distinguishable features in their amino acid sequences suggesting differences in their structures and secretion mechanisms [6, 12]. The possibility that these differences may have ecophysiological implications for N_2_O reduction in diverse environmental settings has recently been a topic of broad interest. Specifically, as N_2_O concentration measured at its source, e.g., agricultural soil and activated sludge, is rarely above low micromolar concentrations, particular attention has been paid to differences in their ability to compete for trace N_2_O [13, 14]. Several previous research results suggested that clade II NosZ generally have higher affinity for N_2_O than clade I NosZ [15–17]. Measurements of whole-cell N_2_O reduction kinetics yielded significantly lower apparent half-saturation constants for the microorganisms harboring clade II *nosZ*, e.g., *Dechloromonas* spp. and *Azospira* spp., than those harboring clade I *nosZ* [16, 18]. The microorganisms possessing clade II *nosZ* were selectively enriched in bioreactors of different configurations where biological reduction of low-concentration N_2_O was observed [19, 20]. In recent experiments that examined the feasibility of removing N_2_O from activated sludge bioreactor exhaust gas via biotrickling filtration, *Flavobacterium* spp., putatively possessing a clade II *nosZ*, showed the highest relative abundance among the biofilm-forming microorganisms [19]. In another recent study where the N_2_O-reducing biofilm was selectively enriched on the membrane surface of a gas-permeable membrane reactor, *Azospira* spp. and *Dechloromonas* spp., putatively possessing clade II *nosZ*, dominated the microbial population [20]. Contradicting observations have also been reported. A long-term chemostat enrichment of an activated sludge-seeded culture with N_2_O as the sole and limiting (i.e., undetectable in the reactor) electron acceptor observed clade I *nosZ* dominating the *nosZ* pool [21]. The inconsistency among these pioneering works may have stemmed from spatially non-uniform distribution of gaseous N_2_O within the reactors, and also possibly from molecular quantitative analyses with reliability and/or resolution issues [10, 17, 22]. Another possible explanation is that the clade I-vs.-clade II distinction may not be sufficient for rationalizing different capabilities of N_2_O-reducing microorganisms to consume trace N_2_O, as several strains of clade I *nosZ*-harboring microorganisms have been found to exhibit whole-cell half-saturation constants (K_m,app_) as low as those of *Azospira* spp. and *Dechloromonas* spp. and/or higher specific affinity $\left( {\alpha _{N2O} = V_m/K_{m,\;app}} \right)$ [23].

An often overlooked environmental factor that may also have caused such conflicting observations is the role of trace O_2_. Many denitrifying and non-denitrifying N_2_O reducers are facultative anaerobes and prefer O_2_ as the electron acceptor over N_2_O when both electron acceptors are available [24]. In most laboratory settings where microbial N_2_O reduction is observed, sub- or low-micromolar O_2_ is often regarded irrelevant, as the amounts or the rates of N_2_O supplied to the experimental cultures far exceed those of O_2_ [17, 20, 21]. Under such conditions, the proportion of microorganisms growing on O_2_ respiration would be negligible relative to those dependent on N_2_O respiration. In environmental systems where N_2_O emission is of great concern, however, O_2_ penetrating into the anoxic niches may be substantially higher in concentration than N_2_O produced in situ or transported from neighboring sources. The dissolved oxygen concentrations in anoxic activated sludge tanks are, in general, orders of magnitudes higher than the dissolved N_2_O concentrations [25, 26]. In agricultural soils, N_2_O reduction would occur most actively where N_2_O is most readily available, i.e., at the oxic-anoxic interface subjected to O_2_ intrusion and frequent oxic-to-anoxic and anoxic-to-oxic transitions [27]. Microorganisms expressing *cbb*_3_-type cytochrome oxidases and/or cytochrome *bd* respiratory oxygen reductases are capable of thriving under such microoxic conditions [28]. Several recent studies suggested transcriptomic evidence of microaerobic respiration and denitrification simultaneous occurring under microoxic conditions; however, how reduction of trace N_2_O and the microbial community responsible for the reaction would be affected by co-occurring microaerobic O_2_ respiration has not yet been explored [29, 30].

In this study, a chemostat reactor was designed for enrichment of microbial consortia capable of reducing N_2_O supplied at a feed rate several orders of magnitude lower than previously applied for enrichment of N_2_O-reducing microorganisms. To ensure spatially uniform N_2_O-limiting condition throughout incubation, the reactor culture was fed N_2_O dissolved in the inflowing medium, instead of gaseous N_2_O previously used for enriching N_2_O-reducing consortia. Unintended O_2_ penetration into the reactor at a rate exceeding the N_2_O feed rates provided a serendipitous opportunity to emulate environments where reduction of trace N_2_O co-occurs with microaerobic respiration. A gene-centric investigation of these microaerobic reactor cultures persistently reducing <50 nM N_2_O was performed via quantitative PCR (qPCR) analyses and *nosZ*-targeting metagenomic analyses. Further, the metatranscriptomes of the reactor microbiomes were analyzed to identify the actively expressed *nosZ* genes among those found with high relative abundance in the gene-centric analyses. Analyses of the functional gene profiles in the reconstructed metagenome assembled genomes (MAGs) and transcription profiling of the MAGs enabled identification of the *nosZ*-possessing minorities that are likely to have substantially contributed to the observed reduction of trace N_2_O. The findings in this study not only substantiate the importance of *Dechloromonas*-like clade II *nosZ* harboring microorganisms as a consequential environmental N_2_O sink, but also provide novel insights into N_2_O reduction occurring in microoxic environments where O_2_ is consistently introduced and consumed.

## Materials and methods

### Operation of chemostat reactors

The activated sludge samples used as the inocula for the reactors were collected from the anoxic compartment of an activate sludge tank at the Daejeon municipal wastewater treatment plant (WWTP; 36°23’5”N 127°24’28”E) immediately before each set of experiments (December 2019 and July 2020). A pre-sterilized 1-L polyethylene bottle was completely filled with the activated sludge sample, capped immediately to minimize oxygen ingress, and transported to the laboratory in a cooler filled with ice. Upon arrival at the laboratory, 100 mL of the sample was subsampled and stored at −20 °C, and the rest was stored at 4 °C.

Three bioreactor experiments were performed. For convenience, the N_2_O-fed chemostat enrichment performed with the December 2019 sample is hereafter referred to as N2OR1 and the repeat of the experiment (with slight modifications to the incubation condition as described below in detail) and the N_2_O-free control experiment performed with the July 2020 sample as N2OR2 and N2FCR, respectively. The modified MR-1 medium, used for all three reactor experiments, contained per liter of double-distilled water, 0.50 g NaCl, 0.29 g KH_2_PO_4_, 0.50 g K_2_HPO_4_, 0.27 g NH_4_Cl, 1 mL of 1000X trace metal solution (Table S1), and 1 mL of 1000X vitamin stock solution (Table S2; added to the autoclaved medium through a 0.22-µm syringe filter) [31]. Sodium acetate was added to a concentration of 5.0 mM as the sole source of electrons and carbon. Acetate has been known to support growth of taxonomically diverse N_2_O-reducing microorganisms and is a non-fermentable substrate [32]. The amount added was stoichiometrically sufficient to ensure excess supply of electron donor throughout incubation, and acetate concentration was measured at the end of each incubation to experimentally verify this electron donor excess.

The reactor system was constructed with a 2-L pyrex glass bottle with a side-port serving as the main chamber for enrichment of microbial culture (Fig. S1). The bottle was capped with a Duran 4-port GL45 connection system (DWK Life Sciences, Wertheim, Germany) and the side-port with a septum-lined cap. For chemostat operation, fresh medium was supplied into the main chamber from a 5-L pyrex glass bottle prepared with a 4 L initial volume of autoclaved acetate-amended MR-1 medium at a flowrate of 42 mL h^−1^ (i.e., a dilution rate of 0.026 h^−1^). The medium bottle was replaced with a new one when the volume of the remaining medium dropped below 500 mL. Waste culture medium was pumped out from the main chamber into a waste collection bottle at the same flowrate as the fresh medium influx. The medium bottle was equilibrated with a gas stream of N_2_ containing 200 ppmv (N2OR1) or 2000 ppmv (N2OR2) (Special Gas, Daejeon, South Korea; these concentrations are nominal values). The reactor culture was mixed with a magnetic stirrer rotated at 200 rpm. A stream of >99.9999% N_2_ gas was passed through the waste collection bottle at a constant flowrate of 200 mL min^−1^ to prevent reverse ingress of contaminating O_2_ into the main chamber. Liquid flow was controlled with a two-channel peristaltic pump (LabSciTech, Corona, CA) and gas flow with gas flowmeters (Dwyer Instrument, Michigan City, IN).

Operation of the two N_2_O-fed reactors, N2OR1 and N2OR2, differed only in the start-up strategy. The N2OR1 reactor was started as a batch reactor fed with continuous stream of N_2_O-containing N_2_ gas. The main chamber was flushed with >99.9999% N_2_ gas until no further decrease in O_2_ concentration was observed, and the gas supply was switched to N_2_ gas containing 200 ppmv N_2_O (Special Gas, Daejeon, South Korea). The source of O_2_ contamination was uncertain, and dissolved O_2_ concentration could not be reduced below 10 μM during the preconditioning procedure. After the headspace N_2_O concentration reached 200 ppmv, the 1600-mL aqueous phase of the reactor was inoculated with 80 mL of the activated sludge sample. After 57 hours of initial incubation as a batch reactor, N2OR1 was transitioned to continuous operation and examined for 98 days and 61 volume changes.

Improvements to the experimental designs for the N2OR2 experiment were based on the findings from the N2OR1 experiment. To measure the O_2_ intrusion rate, the reactor system was abiotically operated prior to inoculation with the liquid and gas compositions and flowrates identical to those used for the chemostat operation that followed. The main chamber and the waste collection bottle were flushed with N_2_ gas and the medium bottle with N_2_ gas containing 2000 ppmv N_2_O, both with the same flowrate (200 mL min^−1^). After the dissolved oxygen concentration in the main chamber had stabilized, the gas flow into and out of the main chamber was shut down and the liquid flow was switched on. The amounts of O_2_ and N_2_O in the main chamber were monitored over next three days to measure the rates at which N_2_O and O_2_ (via contamination) were introduced and find how these values diverged from the calculated rates assuming ideal reactor operation, 2.05 and 0 μmol h^−1^, respectively (See supplementary materials). After inoculation with 80 mL activated sludge, the reactor system was operated and monitored for 101 days through 63 volume changes. In parallel with N2OR2, a control reactor system, N2FCR, was set up with an identical configuration, medium composition, and inoculant, but without N_2_O in the N_2_ gas stream passed through the medium bottle. The operation of N2FCR was halted on day 59 due to atmospheric oxygen contamination caused by a screw cap that had been accidently left loosened after sampling on day 45.

### Analytical methods

The dissolved O_2_ concentrations in the medium bottle and the main chamber of the reactor systems were measured using a FireSting-O2 optical oxygen meter and fiber-optic oxygen sensor spots (Pyroscience, Aachen, Germany) attached on the inner walls of glass bottles (see supplementary information for detailed description of the method). N_2_O concentration was measured by manually injecting headspace gas samples to an HP6890 Series gas chromatograph equipped with a PLOT Q column and an electron capture detector (Agilent, Palo Alto, CA). Calculations involving both aqueous and gaseous phase concentrations used the dimensionless Henry’s law constants (*H*^*cc*^) of 0.594 and 0.032 for N_2_O and O_2_, respectively [33]. Acetate concentration was measured using a Prominence high-performance liquid chromatograph (Shimadzu, Kyoto, Japan) equipped with an Aminex HPX-87H column (Bio-Rad Laboratories, Inc., Hercules, CA) after filtering the sample with a 0.22-μm pore syringe filter (Advantec, Inc., Tokyo, Japan).

### Extraction and sequencing of nucleic acids

Cell suspensions were collected from N2OR1, N2OR2, and N2FCR at varying time points for monitoring of the reactor cultures via quantitative PCR (qPCR) and 16S rRNA gene amplicon sequencing. At each sampling time point, a 15 mL cell suspension was collected and subsequently distributed to three 5 mL microcentrifuge tubes in 4.5-mL aliquots. Cell pellets were collected from these cell suspensions via centrifugation at 15,000 x g, and DNA was extracted using DNeasy PowerSoil Pro Kit (Qiagen, Hilden, Germany), according to the protocol provided by the manufacturer. The V6-V8 region of 16S rRNA gene was amplified with the 926F-1392R primer set (Table S3), and sequencing of the amplicons was performed at Macrogen Inc. (Seoul, South Korea) on the MiSeq platform (Illumina, San Diego, CA). The amplicon sequence data were analyzed using the QIIME2 pipeline as described in detail in supplementary information [34].

Additionally, DNA and total RNA samples were collected at the end of N2OR1 and N2OR2 operations for shotgun metagenome and metatranscriptome analyses, respectively. The cells for extraction of DNA for metagenome analysis were collected on a 0.22-μm membrane filter (MiliporeSigma, Burlington, MA) via vacuum filtration of a 40-mL cell suspension harvested from the reactor. DNA was extracted from the shredded membrane filter paper using DNeasy PowerSoil Pro Kit (Qiagen). For extraction of total RNA for metatranscriptome analysis, another 40-mL cell suspension sample was separately collected and processed. Immediately after extraction from the reactor, the cell suspension was mixed with the same volume of ice-cold methanol (>99.9%; Sigma-Aldrich, St. Louis, MO) for cell fixation and RNA preservation [35]. Total RNA was extracted from the cells collected on the membrane filter, shredded immediately after vacuum filtration with a razor blade treated with RNaseZAP (Sigma-Aldrich). The filter pieces were placed into a 2 mL RNase-free screw cap tube containing 50 mg of 0.1 mm diameter glass beads (Omni International, Kennesaw, GA). The cells were disrupted with a Bead Ruptor Homogenizer (Omni International, Kennesaw, GA) for 5 minutes at the maximum speed. The tube was flash-frozen in liquid nitrogen three times during the homogenization process to prevent warming of its contents. The total RNA was isolated using TRIzol reagent (Ambion, Austin, TX), and purified using RNeasy MinElute Cleanup Kit (Qiagen) [17, 36]. Ribosomal RNA was removed using MICROBExpress Bacterial mRNA Enrichment Kit (Thermo Fisher Scientific, Waltham, MA), and the sequencing library was prepared using TruSeq Stranded mRNA Kit (Illumina) and barcoded using the TruSeq RNA CD Index Kit (Illumina), according to the instruction provided by the manufacturer.

Shotgun sequencing of DNA and RNA-derived sequencing library was performed at Macrogen Inc. (Seoul, South Korea). The metagenomic DNA was sequenced on an HiSeq 4000 platform (Illumina) with a targeted throughput of 10 Gb (N2OR1) or 30 Gb (N2OR2), and the RNA sequencing library was sequenced on a HiSeq X Ten platform (Illumina) with a targeted throughput of 5 Gb (N2OR1) or 10 Gb (N2OR2).

### Quantitative PCR analyses

Time-series qPCR analyses were performed for real-time quantitative monitoring of the relative abundances of *nosZ* genes in the N2OR1, N2OR2, and N2FCR reactor cultures. TaqMan qPCR assays were performed using the pre-designed primers-and-probe sets targeting the 16S rRNA genes, the two clade I *nosZ* groups (NosZG1 and NosZG2) and the two clade II *nosZ* groups (NosZG3 and NosZG5: summarized in Table S3) [17, 37]. Calibration curves for the qPCR were constructed with serial dilutions (10^8^ to 10^1^ copies per µL) of purified TOPO PCR2.1 vectors (Thermo Fisher Scientific, Waltham, MA) containing the respective PCR amplicons as inserts [17]. The detection limits were below 10^1^ copies per µL for all qPCR reactions, as the standard deviations of C_q_ values were less than 0.35 (the average C_q_ value was 33.0) at this plasmid concentration.

### Metagenome data processing

Low-quality reads were removed from the raw metagenomic reads using Trimmomatic v0.36 software with the parameters set as follows: LEADING: 3, TRAILING: 3, SLIDINGWINDOW: 4:15, and MINLEN: 70 [38]. *De novo* assembly of the quality-trimmed reads was performed using metaSPAdes v3.14.0 with default parameters [39]. Potential gene-coding sequences in the assembled contigs were predicted using Prodigal v2.6.3, and the translated amino acid sequences were annotated using DIAMOND blastp v0.9.31.132 against NCBI’s non-redundant (nr) protein database (accessed on 04/15/2020) [40, 41]. For each gene-coding sequence, only the best hit with the highest bitscore was considered in the downstream analyses. The gene-coding sequences were assigned KEGG Orthology (KO) numbers using the GhostKOALA server (https://www.kegg.jp/ghostkoala). The blastp-based functional annotations were cross-checked with KO number assignments [42].

The predicted *nosZ* sequences in N2OR1 and N2OR2 metagenomes were translated in silico and aligned along with 97 full-length reference NosZ sequences from the nr database using MUSCLE v3.8.31 with parameters set to default values (Table S4) [43]. The alignment was uploaded to CIPRES Science Gateway v3.3 (https://www.phylo.org/), and a maximum likelihood phylogenetic tree was constructed using RAxML v8.2.12 (1000 bootstraps) with the protein substitute model and matrix options set to ‘PROTGAMMA’ and ‘AUTO’, respectively [44]. For quantitative comparison with the qPCR data, each phylogenetic group of the metagenome-derived *nosZ* sequences was categorized into one of the four target groups via in silico PCR of its representative sequence using Geneious software v9 (Biomatters, Auckland, New Zealand). The relative abundances of the *nosZ* sequences assigned to each target group were tallied, and the ratios among these tallied relative abundances were compared with the ratios among the NosZG1-5 qPCR counts.

For the N2OR1 culture not subjected to a separate 16S rRNA gene amplicon sequencing analysis, microbial community composition analysis was performed with the 16S rRNA sequences extracted from the endpoint shotgun metagenome data. Putative 16S rRNA reads were extracted from the quality-trimmed reads using Meta-RNA3 with *molecule type* and *e-value* set to “*ssu*” and “1E-10”, respectively [45]. Full-length 16S rRNA genes were reconstructed from these reads using EMIRGE v0.61.0 with SILVA SSU database release 138 as reference [46, 47]. The reconstructed 16S rRNA genes were assigned taxa using RDP classifier v2.10.2 based on SILVA SSU database release 138 with *minimum threshold* set to 0.8 [47, 48].

### Quantitative metagenomic and metatranscriptomic data processing

The quality-trimmed raw metagenomic reads were mapped on to the assembled contigs using the Burrows-Wheeler Aligner (BWA)-MEM algorithm with default parameters [49]. The resulting sequence alignment mapping (sam) files were sorted using SAMtools v1.10, and the length-normalized read coverage of each contig was calculated using BEDTools (v2.26.0) *genomecov* tool [50, 51]. The read coverage values of the contigs were further normalized by the total number of mapped reads in each sample, yielding the reads-per-kilobase-per-million-mapped-reads (RPKM) values. The RPKM value of an entire contig was taken as the relative abundances of the gene-coding sequences of interest found within the contig, as using longer sequences as mapping templates generally yield less-biased coverage data [52].

The transcription levels of the functional genes of interest were estimated by mapping the metatranscriptomic reads onto the gene-coding sequences in the metagenomes. The quality-trimmed metatranscriptomic reads were mapped onto the rRNA database consisting of the short subunit (SSU) and long subunit (LSU) rRNA sequences from SILVA Ref NR 99 database release 138 and 5S rRNA sequences from the Rfam v14.4 database clustered at 98% identity, using the BWA-MEM algorithm [49]. The non-rRNA metatranscriptomic reads, i.e., the reads not mapped onto any of these rRNA sequences, were extracted and organized into a separate fastq format file using SAMtools v1.10 [49, 50]. These reads were mapped on to the gene-coding sequences of interest in the metagenomic contigs, and counting of the reads was performed using RSEM v1.3.3 [53]. The read counts were normalized with the gene lengths and the total number of non-rRNA metatranscriptomic reads for conversion to RPKM values.

### Reconstruction and analyses of Metagenome Assembled Genomes (MAGs)

Metagenome assembled genomes (MAG) were constructed from the shotgun metagenome data. In addition to the contigs generated with metaSPAdes, another set of contigs was constructed via *de novo* assembly using MEGAHIT v1.2.9 with default parameters [54]. The two sets of contigs were processed in parallel, to maximize the number of extractable MAGs. The contigs were binned into MAGs using MetaBAT v2.15 [55]. Prior to binning, contigs with <2 kb length or <2 mean read coverage over the entire length were excluded using the ‘jgi_summarize_bam_contig_depths’ script in MetaBAT and the coverage data were constructed using BWA-MEM [55]. The MAGs were refined using RefineM as follows [56]. 1) The contigs with genomic properties, i.e., GC content, tetranucleotide frequency distribution, and/or mean read coverage substantially deviating from the mean values were filtered out. 2) The gene-coding sequences in the contigs in a MAG were assigned taxonomy using DIAMOND v0.9.31.132 with GTDB protein database release 80 as reference, and obvious outliers were removed using ‘taxon_profile’ and ‘taxon_filter’ scripts in RefineM v0.1.1 with parameters set to default values [56]. 3) The 16S rRNA gene sequences in a MAG were assigned taxonomy using DIAMOND v0.9.31.132 with reference to GTDB SSU database release 80, and the contigs carrying 16S rRNA genes identified as incongruent to the consensus classification of the MAG were screened out using the ‘ssu_erroneous’ script with default parameters. The quality of the MAGs were assessed via the lineage-specific workflow of CheckM v1.1.2, and those with <75% completeness or >10% contamination were excluded [57]. The two sets of remaining MAGs, one from metaSPAdes assembly and the other from MEGAHIT assembly, were pooled, and an average nucleotide identity (ANI) matrix showing pairwise comparisons among the MAGs was constructed using Mash and ANImf algorithms implemented in dRep v2.5.0 [58]. The pairs of MAGs with >99% ANI were dereplicated using the ‘dereplicate’ command in dRep v2.5.0 [58]. The MAGs from N2OR1 and N2OR2 metagenomes, constructed and processed independently up to this stage, were pooled and dereplicated using the same procedure. The read coverages of the finalized set of MAGs were computed by mapping the quality-trimmed reads onto the contigs in the MAG bins using the BWA-MEM algorithm [49].

The taxonomic affiliation of each unique MAG was determined using the Microbial Genomes Atlas (MiGA) server (http://microbial-genomes.org/; accessed on 01/27/2021) [59]. Phylogenetic marker genes were identified in the MAGs using PhyloSift v1.0.1 with markers database 4 (release 2018-02-12) as reference [60]. The phylogenetic relationship among the MAGs was inferred by aligning the translated amino acid sequences of the concatenated marker gene sequences using MUSCLE v3.8.31 with default parameters [43]. After removing poorly aligned regions with Gblocks v0.91b, the alignment file was uploaded on CIPRES Science Gateway v3.3 (https://www.phylo.org/), and a maximum-likelihood phylogenetic tree with 1000 bootstraps was constructed using RAxML v8.2.12 [44, 61].

The transcription profiles of the protein-coding genes of interest in the MAGs were imported from the read count data generated with RSEM. The genes of interest included *nosZ* and other nitrogen-cycling functional genes and *cydAB* and *ccoN*/*ccoO/ccoNO* genes encoding cytochrome *bd* ubiquinol oxidases and *cbb*_3_-type cytochrome *c* oxidases, respectively, putatively involved in high-affinity O_2_ reduction. The median transcript abundance of single-copy marker (SCM) genes that have been reported to be constitutively expressed in bacterial cells served as a measure of overall transcription activity in the microorganism represented by a MAG [62–64]. The list of the analyzed genes is summarized in Table S5. The KO-based annotations were used for reconstruction and transcription profiling of the KEGG pathway modules for the MAGs [65].

## Results

### Operation of the chemostat reactors

Despite the low N_2_O feed rates, biotic N_2_O removal was consistently observed throughout the entire study periods in the N2OR1 and N2OR2 reactors (Fig. S2). The actual N_2_O concentration in the headspace of the N2OR1 reactor after equilibration with N_2_ gas containing 200 ppmv N_2_O plateaued at 230.0 ± 0.3 ppmv (5.61 ± 0.01 μM in the aqueous phase). The dissolved O_2_ concentration was 9.5 ± 0.2 μM. After inoculation, the headspace N_2_O concentration eventually decreased to 109.1 ± 2.6 ppmv after 57 h of batch incubation. After the reactor operation was transitioned to the continuous mode of operation with N_2_O supplied as dissolved in the influent medium, the N_2_O concentration in the headspace of the reactor decreased below the detection limit (<2 ppmv, equivalent to <49 nM in the aqueous phase at 25 °C) within 90 min. Throughout 98 days of continuous operation, N_2_O concentration in the headspace was sustained below the detection limit, implying a constant N_2_O reduction rate of 0.13 μmol L^−1^ h^−1^. During the chemostat operation, dissolved O_2_ concentration hovered between 8.0 μM and 10 μM (9.0 ± 0.4 μM from 33 datapoints collected between day 3 and 98).

The N2OR2 experiment was started with abiotic continuous operation of the reactor, during which the O_2_ penetration rate and N_2_O supply rates were estimated (see supplementary information and Fig. S3). After inoculation with the activated sludge sample at 88 h, the dissolved O_2_ concentration decreased from 71.6 μM to 6.3 μM within 6 h. A rapid decrease in N_2_O concentration followed the drop in O_2_ concentration, and the N_2_O concentration dropped below the detection limit at 100 h. As observed with N2OR1, N_2_O was undetectable throughout 101 days of reactor operation and O_2_ concentration remained nearly constant within 4.0-5.0 μM range (4.8 ± 0.2 μM from 24 datapoints collected between day 5 and 101). Assuming that O_2_ penetration and N_2_O supply rates were constant throughout the reactor operation, the steady-state rates of O_2_ and N_2_O consumption were estimated as 14.3 ± 0.1 μmol L^−1^ h^−1^ and 0.90 ± 0.04 μmol L^−1^ h^−1^, respectively. Acetate concentrations in the reactor effluent measured at the end of incubation were 4.15 ± 0.01 mM and 4.17 ± 0.07 mM for N2OR1 and N2OR2, respectively, indicating that the reactor cultures were not electron donor-limited.

### Microbial community compositions of the reactor cultures

**Fig. 1:**
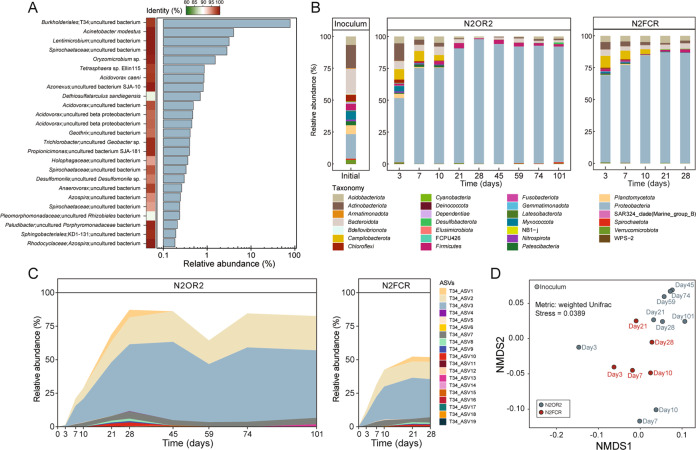
Microbial community composition analyses of the N2OR1, N2OR2, and N2FCR cultures. **A** The taxonomic affiliations and relative abundances of 16S rRNA genes reconstructed from the shotgun sequence reads obtained from metagenome sequencing of the N2OR1 reactor culture collected on day 98. **B** Time series of microbial community compositions of the N2OR2 and N2FCR reactor cultures. **C** The relative abundance of the amplicon sequence variants (ASV) from the N2OR2 and N2FCR reactor cultures with the uncultured strain T34 as the best BLAST hit, i.e., the closest taxon. **D** The nonmetric multidimensional scaling (NMDS) plot of the N2OR2 and N2FCR microbial communities showing the migration of the communities over the course of incubation.

The microbial communities of N2OR1, N2OR2, and N2FCR reactor cultures were invariably dominated by microorganisms belonging to a single taxon affiliated to the *Burkholderiales* order (Fig. 1). The endpoint analysis of the N2OR1 microbial community showed that 29,587 out of 38,727 reads mapped to 74 unique 16S rRNA genes reconstructed from the metagenome were assigned to this taxon, which exhibited 98.4% 16S rRNA gene sequence identity with the uncultured strain T34 (accession number: Z93984.1). Other relatively abundant taxa (>1% relative abundance at genus level) included *Acinetobacter* (3.9%), *Lentimicrobium* (3.3%), *Spirochaetaceae* (3.4%), and *Oryzomicrobium* (1.5%) (Fig. 1A).

The results of the time-series monitoring of the N2OR2 and N2FCR communities also implied that the microoxic conditions of the reactors were largely favorable for the close relatives of the strain T34 (Fig. 1B). The amplicon sequence variants (ASVs) affiliated with this taxon had not been detected in the inoculant, presumably due to their relative scarcity. The microbial community of the inoculum was evenly distributed among the phyla *Actinobacteriota* (18.1%), *Bacteroidota* (19.5%), and *Proteobacteria* (19.2%), and the high Shannon index (8.7) also supported that the microbial population in the inoculum was diverse and evenly distributed (Fig. 1B). The first 28 days of incubation increased the relative abundance of *Proteobacteria* to 97.7% in N2OR2 and 86.6% in N2FCR, reducing the Shannon indices to 2.9 and 4.6, respectively. The cumulative relative abundance of 19 ASVs affiliated to the strain T34 (<7 pairwise mismatches among the 477-bp ASVs) gradually increased to 87.2% and 51.9% of the N2OR2 and N2FCR cultures, respectively (Fig. 1C). *Actinobacteriota* and *Bacteriodota* persisted as significant populations in both reactors throughout the study period. The genus-level taxa apart from the strain T34 with relative abundance higher than 0.5% in the N2OR2 reactor at the end of incubation (day 101) were *Christensenellaceae* R-7 *group*, *Trichlorobacter*, and *Dechloromonas*. Also notable was the transient emergence of *Campilobacteria* (43.9–100% affiliated to *Acrobacter* spp.) as one of the most abundant taxa at the earlier stage of incubation and its eventual disappearance by day 28.

The non-metric multidimensional scaling (NMDS) plot showing migration of the N2OR2 and N2FCR reactor communities clearly illustrates the similarity between progression of enrichment in the two reactors (Fig. 1D). The NMDS plots based on unweighted UniFrac and Blay-Curtis distance metrics, also showed the two reactor communities were heading in the same direction (Fig. S4). This similarity suggests that the selective pressure that shaped the N2OR2 reactor community, and probably the N2OR1 community as well, was not the availability of trace N_2_O as the electron acceptor. Rather, it was more likely that O_2_ penetrating into the reactor was responsible for the overwhelming enrichment of microorganisms affiliated to the strain T34 and shaping of these unique microbial communities. The substantially lowered relative abundance (2.2% relative abundance) of the microorganisms affiliated to the strain T34 in the microbial community of N2FCR culture collected after the accident (day 59) also corroborates that the selective advantage of these uncultured microorganisms was most likely their capability to efficiently utilize low micromolar dissolved O_2_ (Fig. S5)

### Quantitative monitoring of *nosZ* genes in N_2_O-amended and N_2_O-free chemostats

**Fig. 2:**
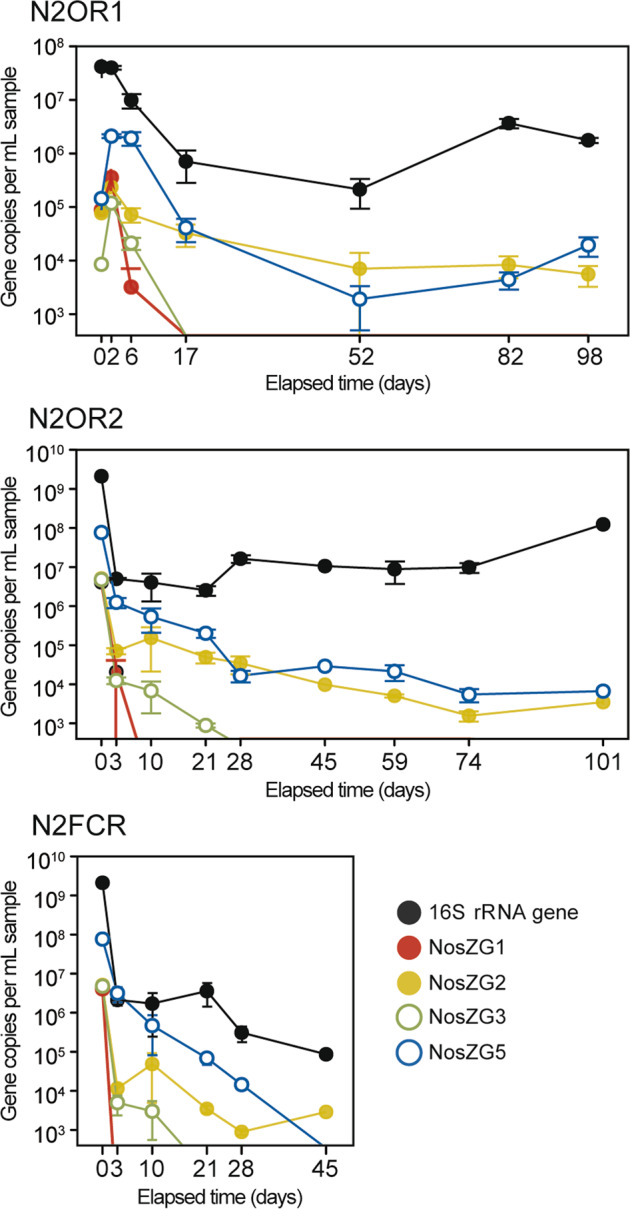
Time series of *nosZ* and 16S rRNA gene copy numbers (per mL sample) in N2OR1, N2OR2, and N2FCR reactor cultures quantified using TaqMan qPCR targeting NosZG1, NosZG2, NosZG3, and NosZG5 groups as well as 16S rRNA gene. The closed and open circles indicate the clade type of each *nosZ* group (closed: clade I, open: clade II).

Monitoring of the four *nosZ* groups (targeted by the NosZG1, NosZG2, NosZG3, and NosZG5 qPCR) in both N2OR1 and N2OR2 reactor cultures identified the NosZG2 (*Acidovorax*-like clade I *nosZ*) and NosZG5 (*Dechloromonas*-like clade II *nosZ*) groups as the most abundant groups of *nosZ* genes (Fig. 2). In N2OR1, the 16S rRNA gene copy number decreased from 4.0 ± 0.3 × 10^7^ to 7.1 ± 4.3 × 10^5^ copies mL^−1^ within 15 days of transition to continuous operation on day 2. In N2OR2 operated without the initial batch incubation, the 16S rRNA gene copy number dropped precipitously after inoculation, down to 5.0 ± 0.3 × 10^6^ copies mL^−1^. These observations imply that the vast majority of cells not capable of growing at a rate higher than the dilution rate (0.026 h^−1^) on the limited supply of O_2_ and/or N_2_O were washed out. Beyond the first two weeks of continuous culture operation, the 16S rRNA gene copy numbers stabilized in the reactors, fluctuating between 2.1 ± 1.2 × 10^5^ and 3.7 ± 0.7 × 10^6^ copies mL^−1^ in the N2OR1 culture (17−98 day) and between 2.5 ± 0.7 × 10^6^ and 1.24 ± 0.03 × 10^8^ copies mL^−1^ (21−101 day) in the N2OR2 culture. The sums of the copy numbers of *nosZ* fluctuated between 9.0 ± 7.0 × 10^3^ and 7.4 ± 2.4  × 10^4^ copies mL^−1^ and 7.4 ± 2.1 × 10^3^ and 2.5 ± 0.5 × 10^5^ copies mL^−1^ in the N2OR1 and N2OR2 cultures, respectively, during the same periods and were orders of magnitude lower than the 16S rRNA gene copy numbers. The sum of the copy numbers of the NosZG2 and NosZG5 target groups was at least 23.2-fold higher than the sum of the other *nosZ* groups at any time point. Further, only NosZG2 and NosZG5 target groups were detectable with qPCR at the end of incubation, i.e., day 98 for N2OR1 and day 101 for N2OR2. As to the comparison between the abundances of NosZG2 and NosZG5, no unequivocal conclusion can be drawn from the qPCR data alone.

The time-series *nosZ* qPCR data from the N2FCR cannot be directly compared with the corresponding data gathered from N2OR1 and N2OR1; however, interesting observations could be gleaned from this data (Fig. 2). While neither NosZG1 nor NosZG3 was detected beyond day 10, the NosZG2 and NosZG5 groups both persisted until day 28, with copy numbers of 8.9 ± 1.5 × 10^2^ copies mL^−1^ and 1.4 ± 0.4 × 10^4^ copies mL^−1^, respectively. The persistence of NosZG2 and NosZG5 populations suggests that at least substantial portions of these *nosZ*-possessing microorganisms grew on microaerobic respiration alone.

### Genomic and transcriptomic profile of *nosZ* in the chemostat cultures consuming trace N_2_O

Metagenomic analyses of the N2OR1 and N2OR2 cultures collected at the end of incubation identified 41 (10 clade I and 31 clade II) and 27 (9 clade I and 18 clade II) unique *nosZ*-coding sequences in the assembled contigs, respectively (Fig. S6). All unique clade I *nosZ* sequences belonged to the NosZG2 target group and 28 out of 49 clade II *nosZ* sequences belonged to the NosZG5 target group. Consistent with the qPCR assays, none were affiliated with *nosZ* of NosZG1, and the NosZG3 group was also underrepresented (7 sequences; Fig. S7).

**Fig. 3:**
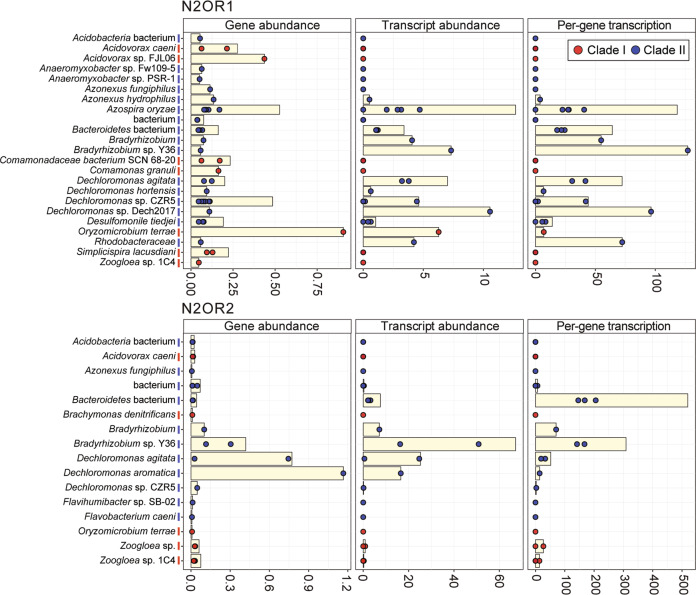
Gene and transcript abundance, and per-gene transcription of *nosZ* in the N2OR1 and N2OR2 reactor cultures collected after 98 and 101 days of chemostat incubation. The bars represent the sum of the abundances of unique *nosZ* sequences (represented as colored dots) affiliated to the taxa. The color of each taxon indicates the *nosZ* clade it belongs to (clade I: red, clade II: blue).

The quantitative compositions of the *nosZ* gene pools were substantially different between the N2OR1 and N2OR2 cultures (Fig. 3). In the N2OR1 culture, the relative abundance of the clade I *nosZ* genes was comparable (2.3 RPKM) to those belonging to the clade II *nosZ* (2.5 RPKM), mostly due to the high abundance of the *nosZ* genes affiliated to *Oryzomicrobium terrae* (0.9 RPKM) and *Acidovorax* spp. (0.7 RPKM). Among clade II *nosZ*, those affiliated to *Dechloromonas* spp. (0.9 RPKM) and *Azospira oryzae* (0.5 RPKM), both grouped with the NosZG5 target group, had the highest relative abundance. Contrastingly, the *nosZ* pool in the N2OR2 was dominated by the clade II *nosZ* genes affiliated to *Bradyrhizobium* spp. (0.5 RPKM) and *Dechloromonas* spp. (2.0 RPKM), together constituting 86.8% of the entire *nosZ* pool in the metagenome. These results were in line with the qPCR-based group-specific quantification of *nosZ*, in that the NosZG2 and NosZG5 target groups were the most abundant groups and also that NosZG2 was the most abundant group in N2OR1 on day 98 while NosZG5 was the most abundant group in N2OR2 on day 101. None of the *nosZ* genes without affiliation to the qPCR target groups had RPKM value above 0.08 in either reactor, confirming that the vast majority of *nosZ* genes were captured with the group-specific qPCR assays.

Metatranscriptomic investigation of the *nosZ* expression profiles clarified that the microorganisms harboring NosZG5 *nosZ* are likely the main players in consumption of trace N_2_O observed in N2OR1 and N2OR2 reactors (Fig. 3). In both N2OR1 and N2OR2, the vast majority of *nosZ* transcripts (47.4 out of 62.3 RPKM total and 116.2 out of 125.3 RPKM total, respectively) were assigned to the NosZG5 target group. More specifically, the *nosZ* transcripts affiliated to *Dechloromonas* spp. (22.8 RPKM), *Azospira oryzae* (12.7 RPKM), and *Bradyrhizobium* spp. (11.4 RPKM) were the most abundant in N2OR1, and those affiliated to *Bradyrhizobium* spp. (74.3 RPKM) and *Dechloromonas* spp. (41.9 RPKM) were the most abundant in N2OR2. The *nosZ* genes affiliated to *Oryzomicrobium terrae*, despite being the most abundant *nosZ* in the N2OR1 metagenome, was transcribed at a much lower level (6.3 RPKM) than the *nosZ* belonging to NosZG5. The clade II *nosZ* gene affiliated to an unidentified *Bacteroidetes* bacterium (7.6 RPKM) was the only transcribed non-NosZG5 *nosZ* over 1 RPKM in N2OR2.

### Analyses of the metagenome-assembled genomes (MAGs)

After quality-screening and dereplication, 14 and 16 MAGs were recovered from the N2OR1 and N2OR2 metagenomes, respectively (Table S6). The genome size (the cumulative length of contigs in a MAG bin) and the GC content of the recovered MAGs ranged between 0.51-1.47 Mbp and 32.0-40.7%, respectively (Table S6). All reconstructed MAGs, with a single exception (MS_R2_24), were assigned taxa at the phylum level. The phyla represented by the MAGs included *Proteobacteria*, *Bacteroidetes*, *Spirochaetes*, *Actinobacteria*, *Firmicutes*, *Acidobacteria*, *Ignavibacteriae*, *Chloroflexi*, and *Chlorobi* (Table S6). In general, the MAGs exhibited low level of similarity to reference genomes in the database; therefore, taxon assignment at genus level was possible only for MS_R1_15 (*Oryzomicrobium*), MH_R1_24 (*Acidovorax*), MH_R1_8 (*Acinetobacter*), and MS_R2_31 (*Azovibrio*). The lowest average amino acid identity (AAI) of the concatenated marker sequences of MAGs to their closest matches in the database was 38.8%, suggesting the phylogenetic novelty of the reconstructed MAGs (Table S6).

**Fig. 4:**
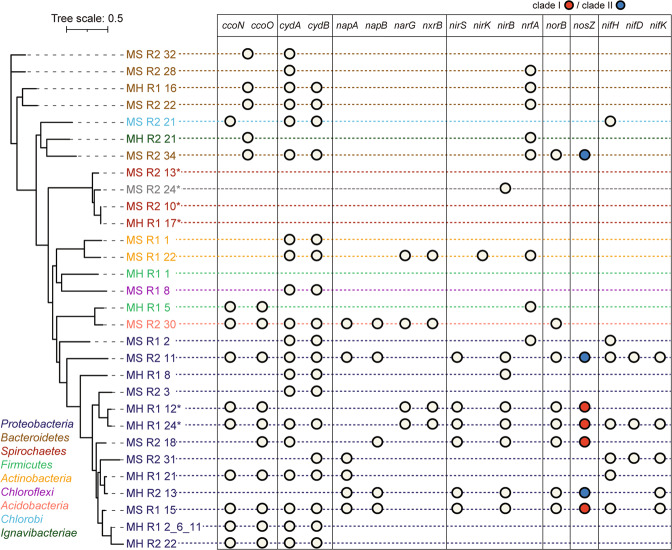
The gene inventories of the MAGs reconstructed from the N2OR1 and N2OR2 metagenomes. MAGs with asterisk (*) are those without any SCM transcript in the metatranscriptome.

Closely related MAGs tended to share similar inventories of the genes involved in nitrogen cycling and oxygen scavenging (Fig. 4). Seven MAGs, three from N2OR1 and four from N2OR2, were found to contain *nosZ* genes (Fig. 4). Two N2OR1 MAGs, MH_R1_24 and MH_R1_12, harbored clade I *nosZ* genes affiliated to the *Comamonadaceae* family (NosZG2). The other *nosZ*-possessing N2OR1 MAG, MS_R1_15, was phylogenetically affiliated to the genus *Oryzomirobium* (NosZG2). The *nosZ* found in this MAG was the most abundant *nosZ* in the N2OR1 metagenome with 95.8% identity at the amino acid level to that of *Oryzomicrobium terrae* (WP_054620228.1). Three clade II *nosZ* genes and a clade I *nosZ* gene were found in the MAGs obtained from N2OR2. The *nosZ* gene binned in MS_R2_11, a MAG taxonomically affiliated to the *Rhizobiales* order of the class *Alphaproteobacteria*, was the highly expressed clade II *nosZ* gene 83.0% identical at amino acid level to *nosZ* of *Bradyrhizobium* sp. Y36 (WP_097659381.1; NosZG5). MH_R2_13, affiliated to the order *Rhodocyclales*, contained a *nosZ* gene with 93.5% AAI to *nosZ* of *Dechloromonas* sp. CZR5 (WP150430259; NosZG5). The concatenated marker sequence of the other MAG with a clade II *nosZ*, MS_R2_34, showed only 40.3% AAI to that of its closest relative in the database (*Algibacter lectus*; GCA_009807485). The *nosZ* in this MAG shared 80.8% AAI with *nosZ* of an uncharacterized bacterium (TRZ66679.1; NosZG3). It should be noted that this MAG was the only one with *nosZ* and *nrfA*, but neither *nirK* nor *nirS*. A MAG affiliated to the order *Rhodocyclales*, MS_R2_18, contained a clade I *nosZ* sharing 91.6% AAI with *nosZ* of *Zoogloea* sp. (KAB2967761.1; NosZG2).

Of the 30 MAGs extracted from the N2OR1 and N2OR2 metagenomes, 24 MAGs included genes encoding either *cbb*_3_-type cytochrome c oxidase (*ccoNO*) or cytochrome *bd* ubiquinol oxidase (*cydAB*), the two terminal oxidases associated with O_2_ scavenging capability under microoxic conditions (Fig. 4). Among the seven *nosZ*-containing MAGs, only MH_R2_13 lacked either terminal oxidase. Most notable of the MAGs possessing *ccoNO* and/or *cydAB* genes were MH_R1_2_6_11 and MH_R2_22 identified as close relatives of the uncultured strain T34. The 16S rRNA gene found in MH_R2_22 was 100% identical to one of the ASVs assigned as the uncultured strain T34 (T34_ASV3 in Fig. 1C). The closest isolated relative of the two MAGs, in terms of the marker sequence identity, was *Hydromonas duriensis* (57.4% and 60.9% AAI with MH_R1_2_6_11 and MH_R2_22, respectively). The two MAGs shared 98.3% average nucleotide identity (ANI), indicating that the two microorganisms represented by these MAGs belong to the same species. Both MAGs had small genome sizes for *Proteobacteria* with 0.6 Mbp and 0.5 Mbp for MH_R1_2_6_11 (from N2OR1) and MH_R2_22 (from N2OR2), respectively, which can hardly be due to incomplete genome assembly, as the genome completeness of MH_R1_2_6_11 and MH_R2_22 were 95.1% and 87.6%, respectively. As anticipated, 87.5% of the N2OR1 reads and 97.7% of the N2OR2 reads covered by the binned contigs mapped onto MH_R1_2_6_11 and MH_R2_22, respectively.

### Transcription of *nosZ* and cytochrome oxidase genes in the MAGs

**Fig. 5:**
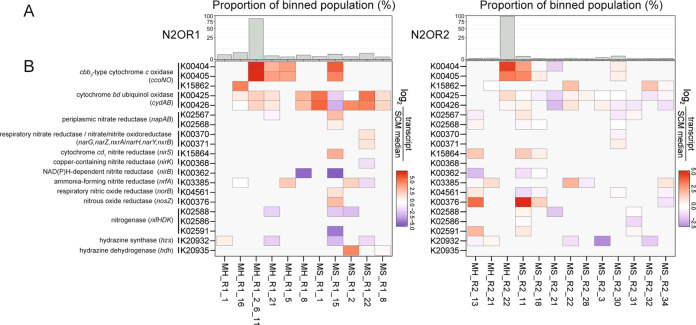
Transcription profiles of the genes encoding nitrogen cycle enzymes and cytochrome oxidases in the MAGs recovered from N2OR1 and N2OR2. **A** Relative genomic abundances of the recovered MAGs. The presented percentage values were calculated by dividing the number of the metagenomic reads mapped onto the MAGs by the total number of short reads mapped onto all binned contigs. **B** Heatmaps showing the transcript abundance of *nosZ*, *ccoNO*, *cydAB*, and other nitrogen-related genes in the MAGs. The MAGs without mapped SCM transcript were excluded.

The genes encoding the cytochrome *bd* ubiquinol oxidase (*cydAB*), *cbb*_3_-type cytochrome *c* oxidase (*ccoNO*), and nitrous reductase (*nosZ*) were among the highly expressed genes in many of the MAGs (Fig. 5). The transcription level of *ccoN* and *ccoO* genes were remarkably high (>11-fold higher than the median transcription level of the single copy marker (SCM) genes) in MH_R1_2_6_11 and MH_R2_22, suggesting that these close relatives of the uncultured strain T34 thrived on O_2_ scavenging. The *nosZ* gene in the *Oryzobacterium*-affiliated MAG, MS_R1_15, was transcribed at a 6-fold higher level than the median of the SCM genes, suggesting that this microorganism may have contributed to removal of trace N_2_O; however, transcription of *ccoN* and *ccoO* genes were several folds higher, suggesting that the main mode of survival for this microorganism was more likely O_2_ scavenging than N_2_O reduction. The two *Acidovorax nosZ* of MH_R1_24 and MH_R1_12 had no mapped transcriptome reads, suggesting that these microorganisms are unlikely to have contributed to the N_2_O reducing activity of the N2OR1 culture. *Dechloromonas nosZ* of MH_R2_13 and *Bradyrhizobium nosZ* of MS_R2_11, both grouped with the NosZG5 target group, showed the highest transcription levels, with 14- and 37-fold higher than the median of the SCM genes in the respective MAGs. As in MAG MS_R1_15, *ccoNO* was relatively highly expressed, suggesting the microorganisms simultaneously utilized O_2_ and N_2_O; however, unlike MS_R1_15, the *nosZ* transcription level in MS_R2_11 was approximately four-fold higher than that of *ccoNO*. The two minor *nosZ*-containing MAGs in N2OR2, MS_R2_18 and MS_R2_34, also showed simultaneous expression of the cytochrome oxidase genes and *nosZ*. These observations suggest that under the conditions where the electron acceptors are scarce, many *nosZ* possessing microorganisms may adopt a strategy of simultaneously utilizing O_2_ and N_2_O.

## Discussion

Identification of the active sinks of N_2_O is crucial for understanding of dynamics and emission of N_2_O in natural and built environments [17, 18, 66, 67]. This study, by combining culture-based experiments with metagenome and metatranscriptome analyses, identified the groups of microorganisms that are likely to function as such crucial N_2_O sinks. The designed N_2_O supply rates to the N2OR1 and N2OR2 reactors were sufficiently low to ensure sub-micromolar steady-state N_2_O concentration and limit N_2_O-dependent microbial growth in the reactors, and provision of N_2_O in dissolved form and constant stirring ensured constant uniform N_2_O limitation in the reactor cultures. Further, operation of the reactors as chemostats ensured that microorganisms with the specific growth rates lower than the dilution rate were eventually washed out [68]. Thus, persistence of N_2_O reduction activity in the reactors attested the existence of microorganisms that can utilize <50 nM dissolved N_2_O as an electron acceptor, corroborating the previous hypothesis that specific guilds of N_2_O reducing microorganisms may have evolved to benefit from trace N_2_O leaked from cohabiting denitrifiers [66, 67].

Penetration of O_2_ into the reactors was unavoidable, and due to the low N_2_O feed rate, dissolved O_2_ concentration in the reactor (9.03 ± 0.42 µM and 4.80±0.22 µM in N2OR1 and N2OR2, respectively) was always orders of magnitude higher than dissolved N_2_O concentration (<50 nM), complicating the search for the microorganisms contributing to high-affinity N_2_O reduction. Despite the overwhelming dominance of the close relatives of the strain T34 in the N_2_O-fed reactors, their participation in N_2_O reduction was ruled out due to the following reason: 1) The N2FCR reactor free of N_2_O was also dominated by this same group of bacteria; 2) the sums of *nosZ* copy numbers, as quantified with the qPCR assays, were orders of magnitudes lower than the 16S rRNA gene copy numbers in the N2OR1 and N2OR2 reactors; and 3) neither of the two MAGs affiliated to the uncultured strain T34 contained any *nosZ* gene.

The results of qPCR and metagenome analyses both suggest that the vast majority of the *nosZ*-possessing microorganisms that avoided washout in the reactors were those with NosZG2 and NosZG5. The qPCR results did not yield conclusive results regarding the quantitative comparison between NosZG2 and NosZG5 in either reactor. However, the metagenome analyses clearly showed NosZG5 being substantially more abundant than NosZG2 in N2OR2, where N_2_O was supplied and consumed at a 7-fold higher rate than N2OR1, suggesting that the microorganisms harboring NosZG5 benefited from their capability to compete for trace N_2_O (Fig. 3). The dominance of NosZG5 in the metatranscriptomic *nosZ* pools (82.8% and 92.4% in N2OR1 and N2OR2, respectively) further suggested that the NosZG5 target group was largely responsible for N_2_O reduction, regardless of their relative abundance. These observations were consistent with the previous measurements of N_2_O consumption kinetics, where *Dechloromonas* spp. and *Azospira* spp., possessing *nosZ* belonging to NosZG5 consistently exhibited the lowest half-saturation constants among diverse N_2_O-reducing microorganisms [16, 18, 69].

What remains uncertain is whether sustenance, i.e., growth at a rate higher than the dilution rate, of N_2_O-consuming microorganisms harboring the NosZG5 group in the chemostats required O_2_ as a supplementary electron acceptor. Selection of the NosZG5 group in N2OR2 suggest that the capability to efficiently utilize submicromolar N_2_O substantially contributed to growth and selection of these microorganisms over other *nosZ*-possessing microorganisms. It should be noted that the recovery of MAG (MH_R2_13) with NosZG5 *nosZ* but neither *ccoNO* nor *cydAB*, was not sufficient to infer the presence of N_2_O reducers entirely independent of O_2_. Rather, the simultaneous elevated transcription of NosZG5 *nosZ*, *ccoNO*, and *cydAB* in the more abundant microorganism represented by MS_R2_11 suggest that reduction of N_2_O was mainly carried out by the microorganisms of NosZG5 simultaneously utilizing O_2_ and N_2_O. The near complete absence of clade II *nosZ* of NosZG3 in N2OR1 and N2OR2 contrasted with the previous reverse transcription (RT)-qPCR analyses of anoxic tank activated sludge samples [17]. Possibly, the microorganisms belonging to NosZG3 may not be as well suited for utilization of micromolar O_2_ as a growth-supporting electron acceptor. Also possible is that the microorganisms possessing NosZG3 and NosZG5 *nosZ* may have distinguishable capability in utilizing trace N_2_O for main or supplementary growth-supporting electron acceptor, even though the two *nosZ* groups have been referred to as a single clade in most literature.

The microorganisms affiliated to the uncultured strain T34 of the order *Burkholderiales* dominated both N2OR1 and N2OR2 cultures, presumably thanks to their capability to scavenge oxygen utilizing the *cbb*_3_-type cytochrome *c* oxidase (encoded by *ccoNO*) [70–72]. Thus, little information could be gathered regarding N_2_O-reducing microorganisms from the 16S rRNA gene amplicon sequencing-based community analyses. Moreover, the abundance of the *nosZ* genes in the metagenomes could not serve as an indicator for estimating the contributions of different *nosZ*-possessing microorganisms to removal of trace N_2_O, due to the enrichment of *nosZ*-possessing microorganisms equipped with *ccoNO* or *cydAB*, as exemplified by the abundance of NosZG2 *nosZ* in the N2OR1 metagenome, which were hardly expressed (Fig. 3). These drawbacks with DNA-based analyses suggest the necessity of RNA- or protein-level investigation for identification of true contributors to N_2_O reduction in environmental microbiomes, as the environmental niches where denitrification occurs are rarely completely segregated from neighboring oxic environments and, as in N2OR1 and N2OR2, O_2_ availability would largely exceed N_2_O availability [25, 73]. The comparison of the qPCR data and the metagenome data confirmed the predictive power of the NosZG1-G5 qPCR that the authors had developed earlier [17].

One of the *nosZ* genes with the highest level of transcription was the gene that showed >83% identity at the amino acid level with the *nosZ* gene found in the genome of an uncharacterized *Bradyrhizobium* isolate (strain Y36; GCF_002531575.1). All other *nosZ* genes in the database affiliated to *Bradyrhizobium* spp. or the order which the genus belongs to, *Rhizobiales*, are classified as clade I; however, this particular *nosZ*, belonged to the NosZG5 target group of clade II, as can be shown in the *nosZ* phylogenetic tree presented in Fig. S6 [74, 75]. Misclassification was unlikely, as the MAG MS_R2_11 containing this gene and the genome of *Bradyrhizobium* sp. Y36 also share high level of similarity (76% ANI). The high abundance of this *nosZ* in both N2OR1 and N2OR2 transcriptomes implies involvement of this atypical member of *Rhizobiales* in high-affinity N_2_O reduction. Additional investigations into the evolutionary history of this *Bradyrhizobium nosZ* may be worthwhile, as the presence of this clade II *nosZ* in *Rhizobiales* may be a rare evidence of horizontal transfer of a *nosZ* gene [76].

Several studies have suggested the potential significance of non-denitrifying N_2_O reducers as environmental N_2_O sinks [6, 10, 74]. The *nosZ* genes affiliated to the microorganisms lacking either *nirK* or *nirS* often constitute substantial portions of agricultural soils’ *nosZ* pools; however, previous attempts to enrich N_2_O-reducing consortia in either suspended-culture or biofilm bioreactors failed to identify such microorganisms as significant constituents [10, 17, 20, 21, 74]. The phylogenetic placements of the recovered *nosZ* genes, as well as the near absence of non-denitrifier *nosZ* in the MAGs, suggest against non-denitrifier N_2_O reducers playing a vital role in the high-affinity N_2_O reduction observed in N2OR1 and N2OR2 reactors. Perhaps, more important than the (*nirK* + *nirS*)/*nosZ* gene ratio in determining N_2_O emissions from anoxic environments where denitrification occurs may be the abundance and activity of the N_2_O-reducing microorganisms capable of sustained consumption of trace N_2_O, as previously suggested [77].

The oxygen levels measured in the reactors may strike many as being too high to be categorized as microoxic. The long-perceived boundary demarcating the O_2_ level that would provide anaerobes selective advantage over aerobes, the ‘Pasteur Point”, is approximately 0.2%, and many biogeochemical models have predicted the critical O_2_ level as low micromolar concentrations (<10 µM) [78–80]. The O_2_ levels observed in N2OR1 and N2OR2 after they had attained pseudo-steady state were consistent with this range of concentrations. However, recent investigations, mostly performed with Unisense microsensors (including the new STOX sensors with improved sensitivity) have reported microbial consumption of O_2_ in the sub-to-low nanomolar concentration range and suggested low-nanomolar concentrations as the critical O_2_ levels for aerobic growth [30, 81, 82]. Given that the typical laboratory strains *Escherichia coli* and *Paracoccus denitrificans* were capable of aerobic growths on nanomolar O_2_, the dominance of the relatives of strain T34 in the reactors and near-absence of such fast-growing heterotrophs in the reactor cultures are difficult to rationalize. Thus, the current study cautiously raises the need to revisit the recent developments in investigation of microbial sub-micromolar O_2_ utilization.

With exception of a few studies where whole-cell Michaelis-Menten kinetics were determined, the N_2_O concentration in the gases fed to N_2_O-reducing microorganisms or consortia in previous laboratory examinations were in the order of 1,000 ppmv or higher [18, 20, 21, 25, 83–85]. Inhibition of N_2_O reductase activity in presence of O_2_ under such laboratory conditions has been widely known; however, not much is known regarding the effect of O_2_ on reduction of N_2_O available at orders-of-magnitude lower concentrations than O_2_, when sub- to low-micromolar concentration ranges are considered [24, 85, 86]. Possibly, the oxygen scavengers utilizing *cbb*_3_-type cytochrome *c* oxidase and/or cytochrome *bd* ubiquinol oxidase may have played a crucial role in sustaining the dissolved O_2_ concentration below the threshold for activation of N_2_O reduction in the N2OR1 and N2OR2 reactors. Micromolar O_2_ may have served as the main growth-supporting electron acceptor for some microorganisms that contributed to N_2_O reduction, as suggested from the transcription profile of MAGs MS_R1_15 (NosZG2), wherein *ccoNO* transcripts were found in substantially higher abundance than *nosZ* transcripts. Further, simultaneous expression of *nosZ* and *ccoNO* observed in MS_R2_11 (NosZG5) with the most highly-expressed *nosZ* in the N2OR2 microbiome suggested that the presence of low-micromolar O_2_ may even be crucial for sustenance of microorganisms that play the most consequential roles in removing trace N_2_O. Evidence of simultaneous expression of *nosZ* and *ccoNO* has also been found in transcriptomes of marine coastal sediments and a sequencing batch reactor during the microoxic phase in recent studies, although measurements of N_2_O concentrations or consumption rates were not accompanied [29, 30]. The interplay between oxygen scavenging and N_2_O reduction may be a crucial underlying mechanism enabling reduction of trace N_2_O at denitrification hotspots, e.g., anoxic tanks in activated sludge WWTP and oxic-anoxic interfaces in agricultural soils, where complete anoxia is rarely attained, keeping N_2_O emissions from these hotspots in check.

## Supplementary information

Supplementary information

## Data Availability

All raw sequence data and the finalized MAGs were deposited in the NCBI database under the BioProject accession number PRJNA789121.
