# Supplementary material for: Identification of nosZ-expressing microorganisms consuming trace N2O in microaerobic chemostat consortia dominated by an uncultured Burkholderiales
Source: ISME J. 2022 Jun 8;16(9):2087–98. doi: 10.1038/s41396-022-01260-5 (PMC9381517; doi:10.1038/s41396-022-01260-5)
Supplement: Supplementary file 1 — Supplementary information [file 41396_2022_1260_MOESM1_ESM.docx]

**Supplementary information**

**Supplementary materials and methods**

**Detailed description of the analytical method used for measurement of dissolved O_2_**

Throughout the study, dissolved O_2_ concentration was measured using FireSting-O_2_ optical oxygen meter and fiber-optic oxygen sensor spots (Pyroscience, Aachen, Germany) attached on the inner walls of glass bottles. The nominal detection range of the sensor, according to the product specification provided by the manufacturer, is 0.3 μM – 1.4 mM. Before each measurement, the optical sensor was calibrated using a two-point method with deionized water equilibrated with atomospheric air at 25 °C (274.1 µM dissolved concentration) and deoxygenated deionized water prepared as follows. A serum bottle (160 mL) containing 98 mL of deionized water was flushed with >99.9999% N_2_ gas for an hour, sealed with an air-tight butyl-rubber stopper (Geo-Microbial Technologies, Ochelata, OK), and autoclaved for 30 minutes. After autoclaving, 1 mL each of 20 mM Na_2_S stock solution and 20 mM L-cysteine stock solution, also prepared in deionized water, flushed with N_2_ for an hours, and autoclaved, was injected into the deoxygenated water, as an effort to minimize dissolved O_2_ [1]. The frequency of the sensor was set to 1 Hz.

**Estimation of rates of O_2_ penetration and N_2_O supply into the N2OR2 reactor culture**

The O_2_ penetration rate and the N_2_O supply rate into N2OR2 were estimated by fitting the experimentally observed N_2_O and O_2_ concentration data to a discretized model assuming quasi-equilibrium between the gaseous and aqueous phases (Equation S1).

$f\left( t+\Delta t \right)= a\Delta t+ f\left( t \right)\left[ 1-\left( \frac{n_{aq}}{n_{aq} + n_{gas}} \right)D(\Delta t) \right]$ (1)

where *f*(*t*) is the total amounts of O_2_ or N_2_O (μmoles) in the reactor vessel (gas plus dissolved) at time *t* (s). *f*(0) was assumed to be zero (i.e., *f*(0) = 0) for either gas. *a* is the rate of O_2_ or N_2_O mass inflow into the reactor (μmol s^-1^), $\Delta t$ is the incremental time (1 s), D is the dilution rate of the reactor (h^-1^), *n_aq_* and *n_gas_* are the amounts of O_2_ or N_2_O (μmoles) in aqueous and gaseous phases of the reactor at time *t*, respectively.

Equation S1 was rewritten to Equation S2, assuming instant quasi-equilibrium between the gaseous and the aqueous phases.

$f\left( t+\Delta t \right)= a\Delta t + f\left( t \right)\left[ 1-\left( \frac{V_{aq}H^{cc}}{V_{aq}H^{cc} + V_{headspace}} \right)D\Delta t \right]$ (2)

where, *V_aq_* and *V_headspace_* are the aqueous working volume (1.6 L) and the gaseous headspace volume (0.74 L), respectively, and *H^cc^* is dimensionless Henry's constant for either of N_2_O or O_2_ (0.594 and 0.032). As the term $\frac{V_{aq}H^{cc}}{V_{aq}H^{cc} + V_{headspace}}$ has a constant value, Equation S2 was further simplified to Equation S3 by substituting the term with a unitless constant *k*.

$f\left( t+\Delta t \right)= a\Delta t + k f\left( t \right)$ (3)

Substitution of $\Delta t$ with 1 s unifies the units of all terms in the equation to μmoles. Thus, Equation S3 can be rewritten as:

$f\left( t+1 \right)= a + k f\left( t \right)$ (4)

*S_t_* is defined as the incremental change in $f\left( t \right)$ over $\Delta t$ (Equation S5).

$S_{t} = f\left( t+1 \right)- f\left( t \right)$ (5)

From Equation S4 and S5, for $t\geq0$

$S_{t+1} = {k S}_{t}$ (6)

Thus the geometric series $\sum_{i=0}^{n} S_{i}$ can be expressed as Equation S7.

$\sum_{i=0}^{n} S_{i} = f(n+1) - f(0)$ (7)

As *f*(0) = 0 and *f*(1) = *a*, for a positive integer *t*

$f(t) = \frac{f(1)(1-k^{t})}{1-k} = \frac{a(1-k^{t})}{1-k}$ (8)

The experimentally determined *k* was fitted to Equation S8 via non-linear regression using *fitModel* function implemented in "mosaic" package in R v4.0.2 [2].

**Analyses of microbial community compositions**

The 16S rRNA amplicon sequence data were analyzed using the QIIME2 pipeline [3]. The raw paired-end 16S rRNA gene amplicon sequence data were converted to QIIME2-compatible datasets. Quality filtering, denoising, chimera removal, and merging of the paired-end sequences were performed using the 'denoise-paired' command implemented in QIIME2, with parameters set to default values. Each unique ASV was assigned taxonomy using a scikit-learn Naive Bayes classifier (‘classify-sklearn’ command) pre-trained with SILVA SSU Ref NR 99 database release 138, with the confidence threshold set to 0.8 [4]. Subsequently, an ASV table was generated by incorporating the taxonomy assignments into the ASV feature table. The ASVs were sorted into phylogenetic groups based on the phylogenetic tree generated with MAFFT and FastTree implemented in QIIME2, both with default parameters [3]. The alpha and beta diversity indices were calculated using the 'diversity alpha-rarefaction' and 'diversity core-metrics-phylogenetic' commands in QIIME2.

**Supplementary results**

**Rates of O_2_ penetration and N_2_O supply into the N2OR2 reactor**

The actual O_2_ penetration rate and N_2_O supply rate calculated using the concentration data collected during abiotic start-up period of the N2OR2 reactor operation showed that O_2_ was available to the reactor culture at a level far higher than N_2_O (Fig. S3). The dissolved O_2_ concentration increased from 4.8 μM to 71.6 μM between 16 h and 88 h and the O_2_ penetration rate estimated by curve-fitting of the data points was 22.9±0.2 μmoles h^-1^. The N_2_O concentration increased from 0 to 705.9±13.6 ppmv in the reactor headspace (17.2±0.3 μM in the aqueous phase assuming equilibrium) during the same period. This headspace N_2_O concentration was substantially lower than the design value, ~1320 ppmv. Thus, the N_2_O feed rate into the reactor calculated from these start-up data, 1.44±0.06 μmoles h^-1^, was approximately 70% of the design N_2_O feed rate (2.08 μmoles h^-1^), presumably due to uncertainties associated with the low fluxes and concentrations.

**Supplementary figures**

**
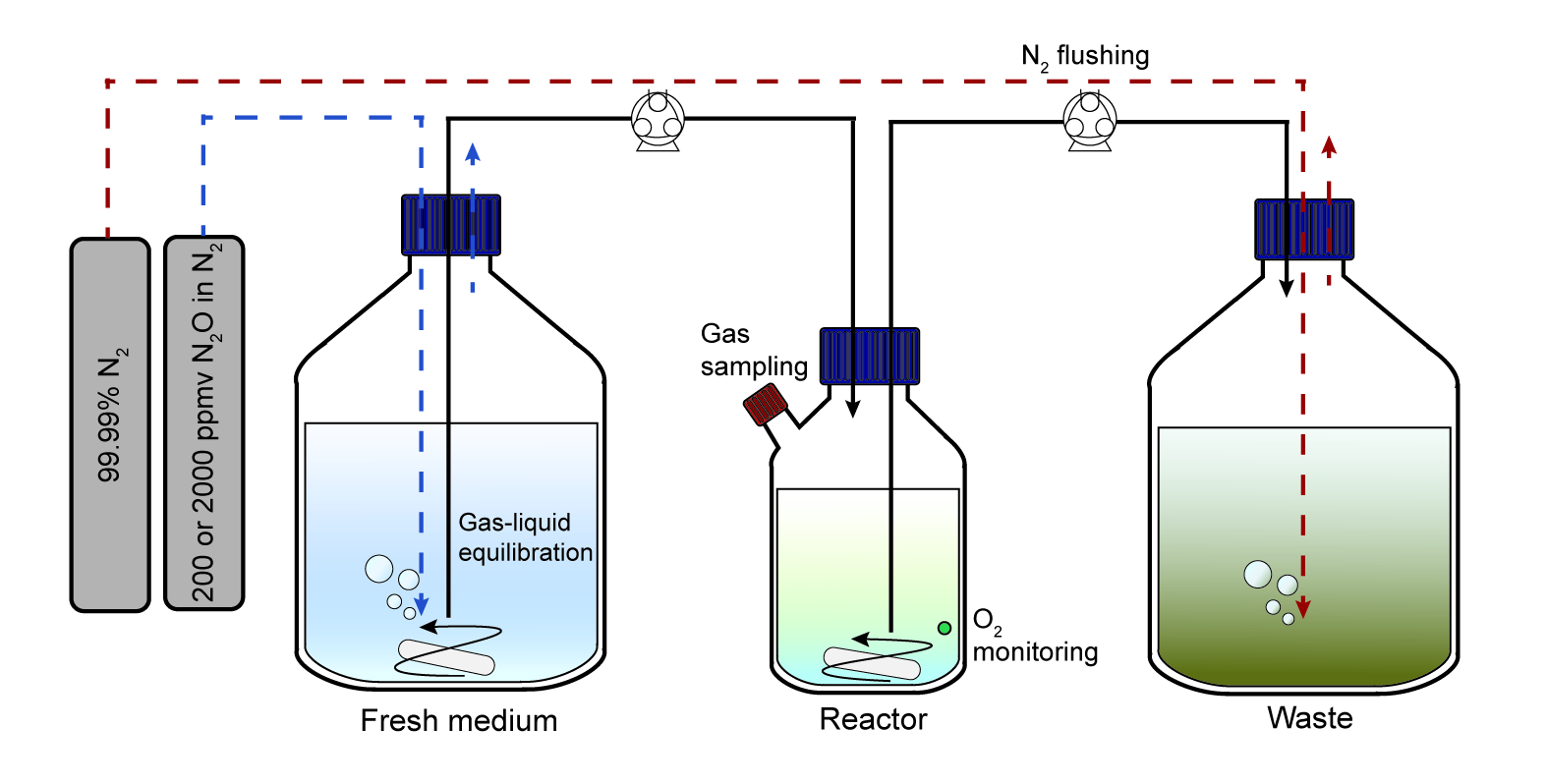
**

**Fig. S1** Schematic diagram of the continuous chemostat reactors (N2OR1 and N2OR2) used for enrichment of the organisms capable of utilizing submicromolar N_2_O. The exactly same setup was used for the N2OR1 (200 ppmv N_2_O) and N2OR2 (2000 ppmv N_2_O) experiments.


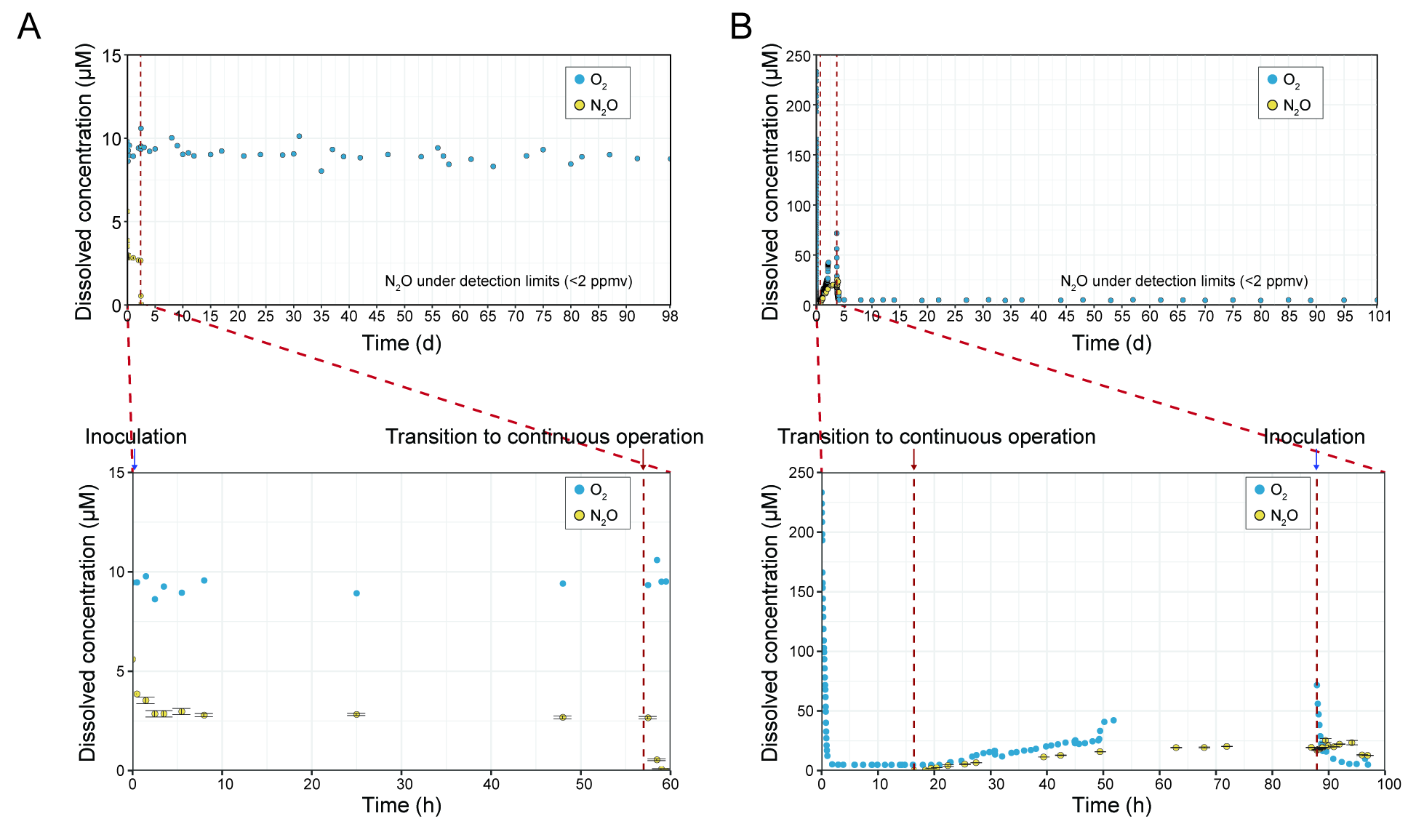


**Fig. S2** Monitoring of O_2_ and N_2_O concentrations in the chemostat reactors (A) N2OR1 and (B) N2OR2. The bottom panels are blown-up figures showing the start-up phases of the reactors in detail. The N2OR1 reactor was inoculated at 0 h and operated as a batch reactor fed a continuous stream of 200 ppmv N_2_O (in N_2_) gas for 57 hours before being transitioned to continuous operation. The N2OR2 reactor was initially operated without inoculation. The system was initially flushed with N_2_ gas for 16 hours to strip oxygen (0 - 16 h) and transitioned to continuous operation for determination of the O_2_ penetration rate and the N_2_O supply rate. The reactor was inoculated at 88 h. Each point representing an O_2_ concentration is the average of 600 measurements taken with the O2 sensor (1 Hz). The error bars for N_2_O concentrations represent the standard deviations of three measurements taken with 5-min intervals.


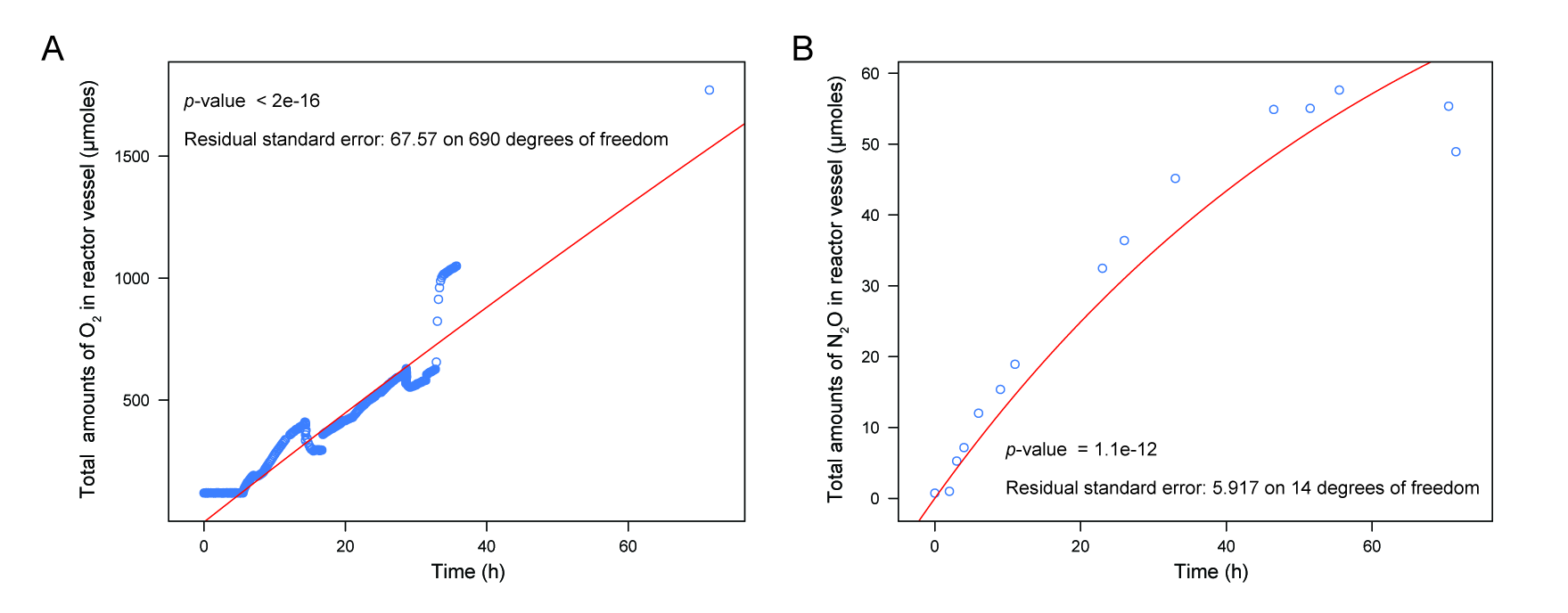


**Fig. S3** Curve-fitting via non-linear regression of the O_2_ and N_2_O data collected during abiotic operation of N2OR2 (16 - 88 h in Fig. S2). The O_2_ penetration rate and N_2_O supply rate into the N2OR2 reactor were estimated by fitting the experimental data to the equation 8 above.


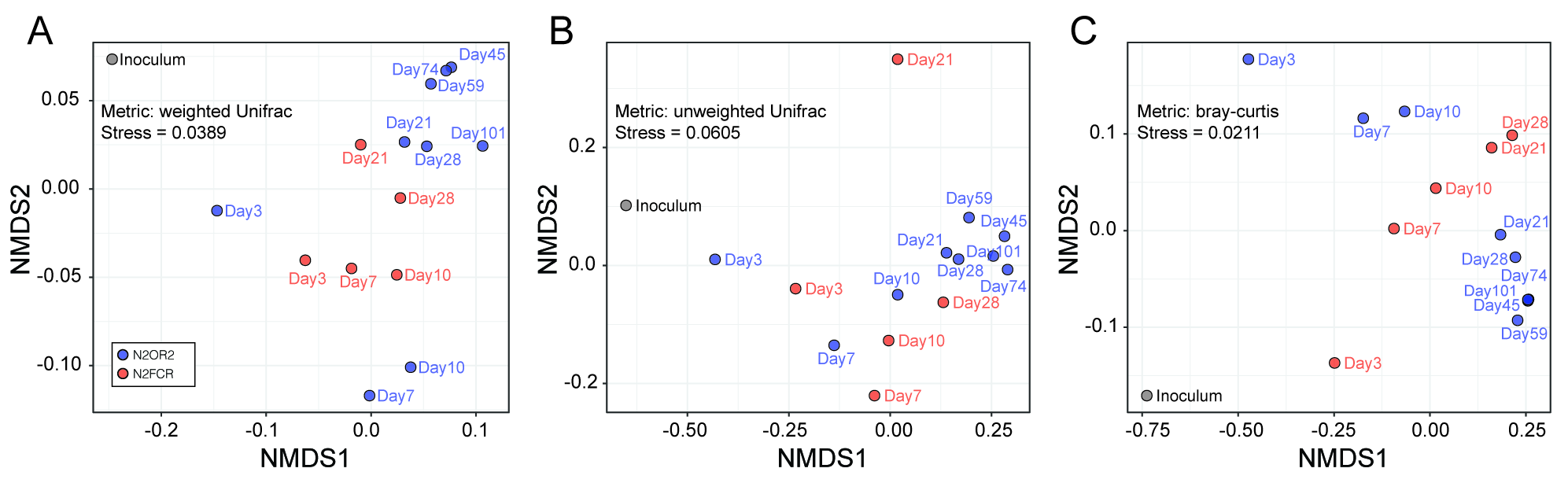


**Fig. S4** NMDS plots showing migration of microbial communities in N2OR2 and N2FCR, constructed based on (A) weighted Unifrac (shown as Figure 1D in the main text), (B) unweighted Unifrac, and (C) Bray-Curtis dissimilatory metrics.


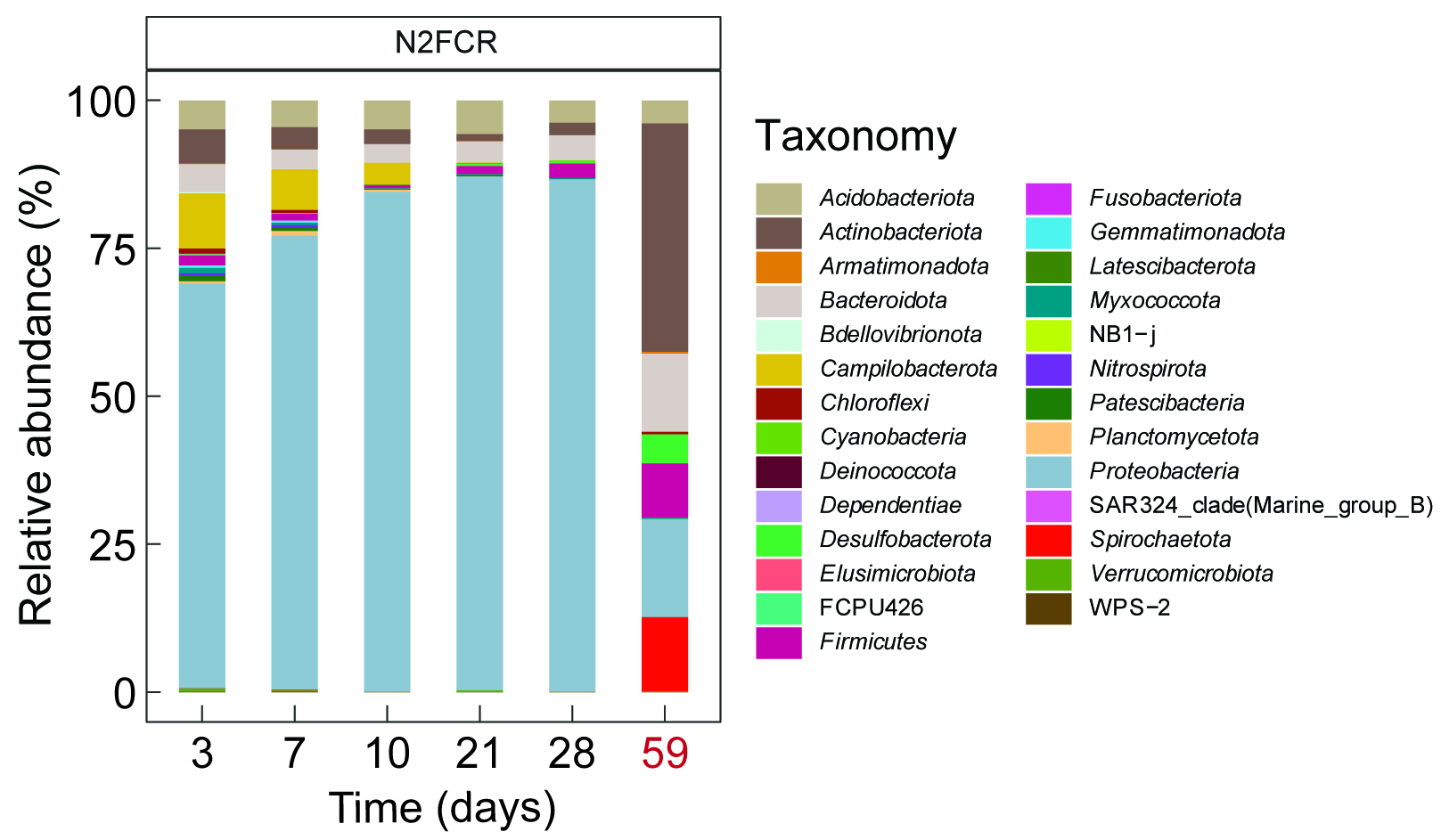


**Fig. S5** The microbial community of the N2FCR reactor culture before (day 3-28 as presented in Fig. 1b) and after (day 59) the accidental O_2_ contamination event that occurred between day 45 and 59. Although not shown in this phylum-level data, the taxa closely affiliated to the uncultured strain T34 of the *Burkholderiales* family was reduced to 2.2% relative abundance in the sample collected on day 59.


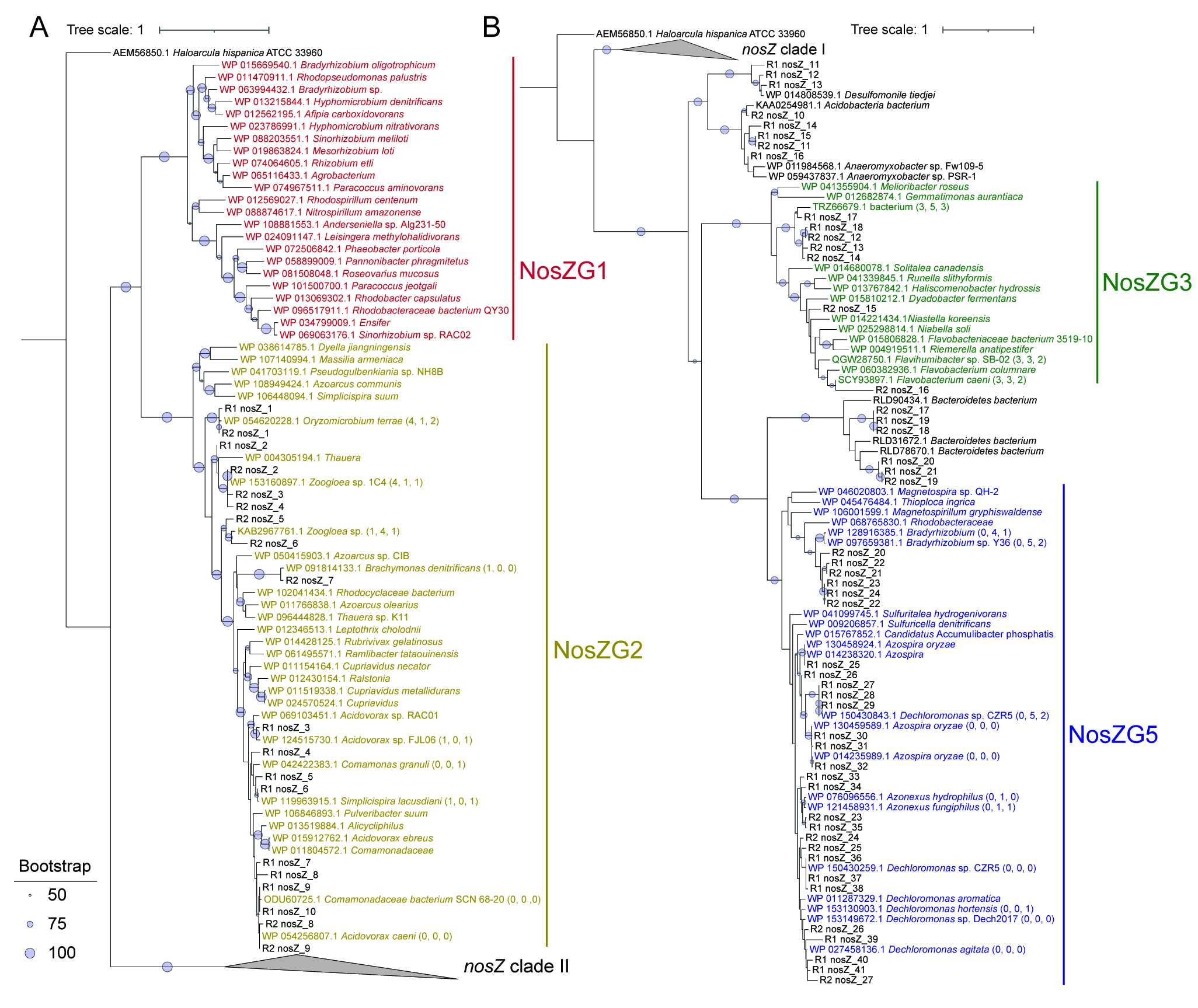


**Fig. S6** Maximum-likelihood tree constructed with the *in silico* translated *nosZ* sequences recovered from the shotgun metagenomes of the N2OR1 (day 98) and N2OR2 (day 101) reactor cultures and the reference NosZ sequences downloaded from NCBI’s nr database. (A) Clade I and (B) clade II *nosZ* are shown separately for readers’ convenience. The color of a leaf indicates its affiliation to a qPCR target group (NosZG1: red, NosZG2: yellow, NosZG3: green, and NosZG5: blue). The three numbers in a parenthesis show the numbers of mismatches to the forward primer, the probe, and the reverse primer, in the order given. The colored leaves without parenthesis are *nosZ* sequences previously reported to be detectable by NosZG1-5 qPCR. Bifurcations with bootstrap values over 50% are indicated with open circle symbols drawn to scale.


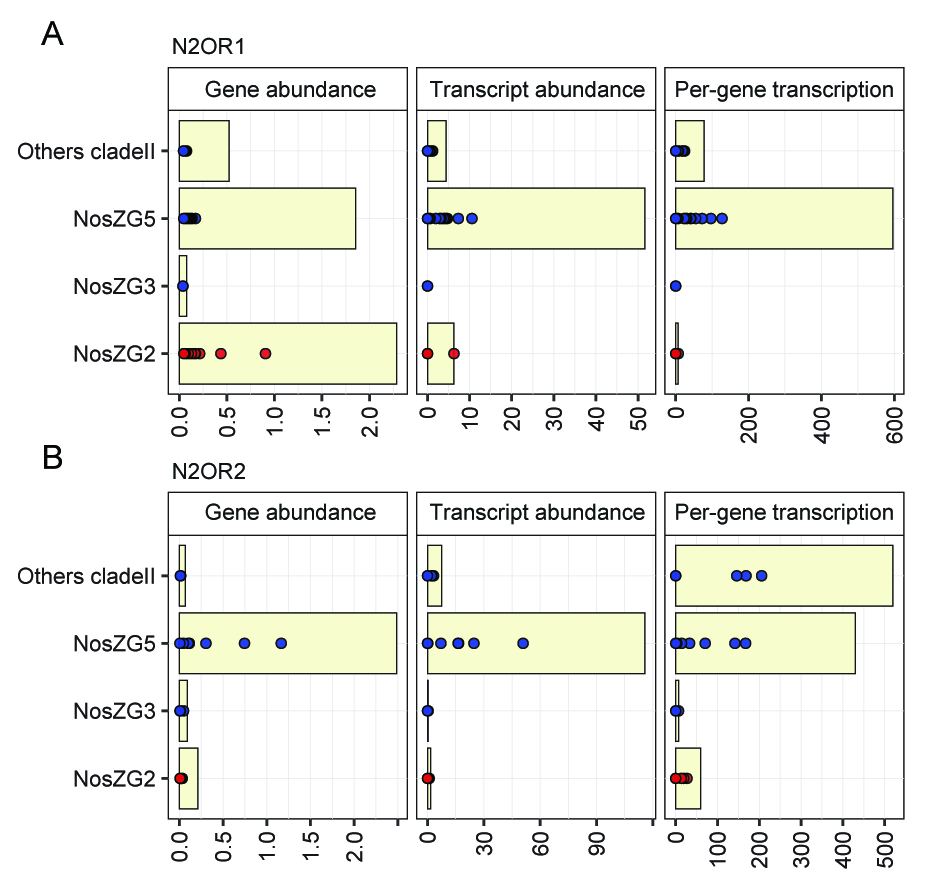


**Fig. S7** Gene and transcript abundance and per-gene transcription of the *nosZ* genes in (A) N2OR1 and (B) N2OR2 metagenomes categorized into the qPCR target groups NosZG2-G5 based on *in silico* PCR of their representative sequences. The NosZG1 target group was omitted, as no reconstructed *nosZ* sequence belonged to the group. The clade II *nosZ* groups with their representative sequence not captured by any *in silico* PCR were categorized into the ‘Other clade II’ group.

**Supplementary tables**

**Table S1** Recipe for the 1000X trace metal solution.

| Elements | Amounts |
| --- | --- |
| CoCl_2_ x 2 H_2_O | 50 mg |
| FeCl_2_ x H_2_O | 35 mg |
| MnSO_4_ x H_2_O | 125 mg |
| CuSO_4_ x 5 H_2_O | 5 mg |
| Na_2_MoO_4_ x 2 H_2_O | 5 mg |
| CaCl_2_ x 2 H_2_O | 50 mg |
| ZnCl_2_ x 2 H_2_O | 25 mg |
| AlK(SO_4_)_4_ x 12 H_2_O | 10 mg |
| H_3_BO_3_ | 5 mg |
| Na_2_WO_4_ x 2 H_2_O | 2.5 mg |
| NiCl_2_ x 2 H_2_O | 10 mg |
| H_2_SeO_3_ | 25 mg |
| ddH_2_O | 100 ml |

**Table S2** Recipe for the 1000X vitamin stock solution.

| Elements | Amounts |
| --- | --- |
| 4-aminobenzoic acid | 50 mg |
| D+-biotin | 20 mg |
| Nicotinic acid | 50 mg |
| Ca-pantothenate | 50 mg |
| Pyridoxine-HCl | 100 mg |
| Folic acid | 20 mg |
| Alpha lipoic acid | 50 mg |
| Thiamine-HCl | 50 mg |
| Cyanocobalamine | 50 mg |
| ddH_2_O | 100 ml |

**Table S3. Primers and probes used for quantitative PCR analyses**

| **Target genes** | **Primers/probe** | **Sequence (5’ → 3’)** | **Slope** | ***y*-intercept** | **Efficiency (%)** | **Temperature cycle** | **Reference** |
| --- | --- | --- | --- | --- | --- | --- | --- |
| Eubacterial 16S rRNA gene | Bac1055YF | ATG GYT GTC GTC AGC T | -3.36 | 36.95 | 98.63 | (95°C, 10 min)  (95°C, 30 s; 56 °C, 1 min; 72°C, 1min) x 40 | Ritalahti et al., 2006 [5] |
|  | Bac1392R | ACG GGC GGT GTG TAC |  |  |  |  |  |
|  | Bac1115Probe | FAM-CAA CGA GCG CAA CCC-TAMRA |  |  |  |  |  |
| NosZG1 | NosZG1F | AAG GTN CGB GTN TAC ATG | -3.19 | 35.89 | 105.9 | (95°C, 5 min)  (95°C, 30 s; 58°C, 1 min; 72°C, 1 min) x 30  (72°C, 5 min) | Kim et al., 2020 [6] |
|  | NosZG1R | CSN NCA TYT CCA TGT GCA |  |  |  |  |  |
|  | NosZG1P | FAM-ACT GCM VBT GGT TCT GCC AYG C-MGBNFQ |  |  |  |  |  |
| NosZG2 | NosZG2F | GRC ATC WKC MMC GAC AAG | -3.37 | 38.8 | 98.0 | (95°C, 5 min)  (95°C, 30 s; 58°C, 1 min; 72°C, 1 min) x 30  (72°C, 5 min) | Kim et al., 2020 [6] |
|  | NosZG2R | HYC TCG RYG TTG TAC TGG |  |  |  |  |  |
|  | NosZG2P | FAM-ACC ACS CGC GTG TTC TGC G-MGBNFQ |  |  |  |  |  |
| NosZG3 | NosZG3F | CAY TTT GCW CCD GAY AAT ATT GAA | -3.65 | 40.19 | 88.1 | (95°C, 5 min)  (95°C, 30 s; 58°C, 1 min; 72°C, 1 min) x 30  (72°C, 5 min) | Kim et al., 2020 [6] |
|  | NosZG3R | BSH WGT TTC ACC BGG CAT |  |  |  |  |  |
|  | NosZG3P | FAM-AAY YTR GAA CAA GAY TGG GAT GTA CCK C-MGBNFQ |  |  |  |  |  |
| NosZG5 | NosZG5F | AAC GAC AAG KCS AAY CCG | -3.34 | 40.19 | 99.4 | (95°C, 5 min)  (95°C, 30 s; 58°C, 1 min; 72°C, 1 min) x 30  (72°C, 5 min) | Kim et al., 2020 [6] |
|  | NosZG5R | GCG GTC GAA CTT CCA GTA |  |  |  |  |  |
|  | NosZG5P | FAM-GCS GTG MTC GAY CTG CGB G-MGBNFQ |  |  |  |  |  |
| V6-V8 of 16S rRNA gene for sequencing | 16S-926Fi5 | TCGGCAGCGTCAGATGTGTATAAGAGACAGAAACTYAAKGAATTGACGG | - | - | - | - | Kim et al., 2020 [6] |
|  | 16S-1392R | GTCTCGTGGGCTCGGAGATGTGTATAAGAGACAGACGGGCGGTGTGTRC |  |  |  |  |  |

**Table S4 The reference *nosZ* sequences downloaded from NCBI’s nr database for phylogenetic analyses**

| Accession number | Protein annotation |
| --- | --- |
| AEM56850.1 | nitrous-oxide reductase [Haloarcula hispanica ATCC 33960] |
| KAA0254981.1 | Sec-dependent nitrous-oxide reductase [Acidobacteria bacterium] |
| KAB2967761.1 | nitrous-oxide reductase [Zoogloea sp.] |
| ODU60725.1 | nitrous-oxide reductase [Comamonadaceae bacterium SCN 68-20] |
| QGW28750.1 | Sec-dependent nitrous-oxide reductase [Flavihumibacter sp. SB-02] |
| RLD31672.1 | Sec-dependent nitrous-oxide reductase [Bacteroidetes bacterium] |
| RLD78670.1 | Sec-dependent nitrous-oxide reductase [Bacteroidetes bacterium] |
| RLD90434. | Sec-dependent nitrous-oxide reductase [Bacteroidetes bacterium] |
| SCY93897.1 | nitrous-oxide reductase [Flavobacterium caeni] |
| TRZ66679.1 | Sec-dependent nitrous-oxide reductase [bacterium] |
| WP_004305194.1 | MULTISPECIES: TAT-dependent nitrous-oxide reductase [Thauera] |
| WP_004919511.1 | Sec-dependent nitrous-oxide reductase [Riemerella anatipestifer] |
| WP_009206857.1 | Sec-dependent nitrous-oxide reductase [Sulfuricella denitrificans] |
| WP_011154164.1 | nitrous-oxide reductase [Cupriavidus necator] |
| WP_011287329.1 | Sec-dependent nitrous-oxide reductase [Dechloromonas aromatica] |
| WP_011470911.1 | TAT-dependent nitrous-oxide reductase [Rhodopseudomonas palustris] |
| WP_011519338.1 | nitrous-oxide reductase [Cupriavidus metallidurans] |
| WP_011766838.1 | nitrous-oxide reductase [Azoarcus olearius] |
| WP_011804572.1 | MULTISPECIES: nitrous-oxide reductase [Comamonadaceae] |
| WP_011984568.1 | Sec-dependent nitrous-oxide reductase [Anaeromyxobacter sp. Fw109-5] |
| WP_012346513.1 | nitrous-oxide reductase [Leptothrix cholodnii] |
| WP_012430154.1 | MULTISPECIES: nitrous-oxide reductase [Ralstonia] |
| WP_012562195.1 | TAT-dependent nitrous-oxide reductase [Afipia carboxidovorans] |
| WP_012569027.1 | TAT-dependent nitrous-oxide reductase [Rhodospirillum centenum] |
| WP_012682874.1 | Sec-dependent nitrous-oxide reductase [Gemmatimonas aurantiaca] |
| WP_013069302.1 | TAT-dependent nitrous-oxide reductase [Rhodobacter capsulatus] |
| WP_013215844.1 | nitrous-oxide reductase [Hyphomicrobium denitrificans] |
| WP_013519884.1 | MULTISPECIES: nitrous-oxide reductase [Alicycliphilus] |
| WP_013767842.1 | Sec-dependent nitrous-oxide reductase [Haliscomenobacter hydrossis] |
| WP_014221434.1 | Sec-dependent nitrous-oxide reductase [Niastella koreensis] |
| WP_014235989.1 | Sec-dependent nitrous-oxide reductase [Azospira oryzae] |
| WP_014238320.1 | MULTISPECIES: Sec-dependent nitrous-oxide reductase [Azospira] |
| WP_014428125.1 | nitrous-oxide reductase [Rubrivivax gelatinosus] |
| WP_014680078.1 | Sec-dependent nitrous-oxide reductase [Solitalea canadensis] |
| WP_014808539.1 | Sec-dependent nitrous-oxide reductase [Desulfomonile tiedjei] |
| WP_015669540.1 | TAT-dependent nitrous-oxide reductase [Bradyrhizobium oligotrophicum] |
| WP_015767852.1 | Sec-dependent nitrous-oxide reductase [Candidatus Accumulibacter phosphatis] |
| WP_015806828.1 | Sec-dependent nitrous-oxide reductase [Flavobacteriaceae bacterium 3519-10] |
| WP_015810212.1 | Sec-dependent nitrous-oxide reductase [Dyadobacter fermentans] |
| WP_015912762.1 | nitrous-oxide reductase [Acidovorax ebreus] |
| WP_019863824.1 | nitrous-oxide reductase [Mesorhizobium loti] |
| WP_023786991.1 | TAT-dependent nitrous-oxide reductase [Hyphomicrobium nitrativorans] |
| WP_024091147.1 | TAT-dependent nitrous-oxide reductase [Leisingera methylohalidivorans] |
| WP_024570524.1 | MULTISPECIES: nitrous-oxide reductase [Cupriavidus] |
| WP_025298814.1 | Sec-dependent nitrous-oxide reductase [Niabella soli] |
| WP_027458136.1 | Sec-dependent nitrous-oxide reductase [Dechloromonas agitata] |
| WP_034799009.1 | MULTISPECIES: TAT-dependent nitrous-oxide reductase [Ensifer] |
| WP_038614785.1 | nitrous-oxide reductase [Dyella jiangningensis] |
| WP_041099745.1 | Sec-dependent nitrous-oxide reductase [Sulfuritalea hydrogenivorans] |
| WP_041339845.1 | Sec-dependent nitrous-oxide reductase [Runella slithyformis] |
| WP_041355904.1 | Sec-dependent nitrous-oxide reductase [Melioribacter roseus] |
| WP_041703119.1 | nitrous-oxide reductase [Pseudogulbenkiania sp. NH8B] |
| WP_042422383.1 | nitrous-oxide reductase [Comamonas granuli] |
| WP_045476484.1 | Sec-dependent nitrous-oxide reductase [Thioploca ingrica] |
| WP_046020803.1 | Sec-dependent nitrous-oxide reductase [Magnetospira sp. QH-2] |
| WP_050415903.1 | nitrous-oxide reductase [Azoarcus sp. CIB] |
| WP_054256807.1 | nitrous-oxide reductase [Acidovorax caeni] |
| WP_054620228.1 | nitrous-oxide reductase [Oryzomicrobium terrae] |
| WP_058899009.1 | TAT-dependent nitrous-oxide reductase [Pannonibacter phragmitetus] |
| WP_059437837.1 | Sec-dependent nitrous-oxide reductase [Anaeromyxobacter sp. PSR-1] |
| WP_060382936.1 | Sec-dependent nitrous-oxide reductase [Flavobacterium columnare] |
| WP_061495571.1 | nitrous-oxide reductase [Ramlibacter tataouinensis] |
| WP_063994432.1 | TAT-dependent nitrous-oxide reductase [Bradyrhizobium sp.] |
| WP_065116433.1 | MULTISPECIES: TAT-dependent nitrous-oxide reductase [Agrobacterium] |
| WP_068765830.1 | MULTISPECIES: Sec-dependent nitrous-oxide reductase [Rhodobacteraceae] |
| WP_069063176.1 | TAT-dependent nitrous-oxide reductase [Sinorhizobium sp. RAC02] |
| WP_069103451.1 | nitrous-oxide reductase [Acidovorax sp. RAC01] |
| WP_072506842.1 | TAT-dependent nitrous-oxide reductase [Phaeobacter porticola] |
| WP_074064605.1 | TAT-dependent nitrous-oxide reductase [Rhizobium etli] |
| WP_074967511.1 | TAT-dependent nitrous-oxide reductase [Paracoccus aminovorans] |
| WP_076096556.1 | Sec-dependent nitrous-oxide reductase [Azonexus hydrophilus] |
| WP_081508048.1 | TAT-dependent nitrous-oxide reductase [Roseovarius mucosus] |
| WP_088203551.1 | nitrous-oxide reductase [Sinorhizobium meliloti] |
| WP_088874617.1 | TAT-dependent nitrous-oxide reductase [Nitrospirillum amazonense] |
| WP_091814133.1 | nitrous-oxide reductase [Brachymonas denitrificans] |
| WP_096444828.1 | nitrous-oxide reductase [Thauera sp. K11] |
| WP_096517911.1 | TAT-dependent nitrous-oxide reductase [Rhodobacteraceae bacterium QY30] |
| WP_097659381.1 | Sec-dependent nitrous-oxide reductase [Bradyrhizobium sp. Y36] |
| WP_101500700.1 | TAT-dependent nitrous-oxide reductase [Paracoccus jeotgali] |
| WP_102041434.1 | nitrous-oxide reductase [Rhodocyclaceae bacterium] |
| WP_106001599.1 | Sec-dependent nitrous-oxide reductase [Magnetospirillum gryphiswaldense] |
| WP_106448094.1 | nitrous-oxide reductase [Simplicispira suum] |
| WP_106846893.1 | nitrous-oxide reductase [Pulveribacter suum] |
| WP_107140994.1 | nitrous-oxide reductase [Massilia armeniaca] |
| WP_108881553.1 | TAT-dependent nitrous-oxide reductase [Anderseniella sp. Alg231-50] |
| WP_108949424.1 | nitrous-oxide reductase [Azoarcus communis] |
| WP_119963915.1 | nitrous-oxide reductase [Simplicispira lacusdiani] |
| WP_121458931.1 | Sec-dependent nitrous-oxide reductase [Azonexus fungiphilus] |
| WP_124515730.1 | nitrous-oxide reductase [Acidovorax sp. FJL06] |
| WP_128916385.1 | MULTISPECIES: Sec-dependent nitrous-oxide reductase [Bradyrhizobium] |
| WP_130458924.1 | Sec-dependent nitrous-oxide reductase [Azospira oryzae] |
| WP_130459589.1 | Sec-dependent nitrous-oxide reductase [Azospira oryzae] |
| WP_150430259.1 | Sec-dependent nitrous-oxide reductase [Dechloromonas sp. CZR5] |
| WP_150430843.1 | Sec-dependent nitrous-oxide reductase [Dechloromonas sp. CZR5] |
| WP_153130903.1 | Sec-dependent nitrous-oxide reductase [Dechloromonas hortensis] |
| WP_153149672.1 | Sec-dependent nitrous-oxide reductase [Dechloromonas sp. Dech2017] |
| WP_153160897.1 | nitrous-oxide reductase [Zoogloea sp. 1C4] |

**Table S5** List of the single-copy marker (SCM) genes used for normalization of transcription data

| KO numbers | Gene name | Protein name |
| --- | --- | --- |
| K06942 | *ychF* | ribosome-binding ATPase |
| K01889 | *pheS* | phenylalanyl-tRNA synthetase alpha chain |
| K01887 | *argS* | arginyl-tRNA synthetase |
| K01885 | *serS* | seryl-tRNA synthetase |
| K01883 | *cysS* | cysteinyl-tRNA synthetase |
| K01869 | *leuS* | leucyl-tRNA synthetase |
| K01883 | *valS* | valyl-tRNA synthetase |
| K01409 | *tsaD* | N6-L-threonylcarbamoyladenine synthase |
| K03106 | *ffh* | signal recognition particle subunit SRP54 |
| K03110 | *ftsY* | fused signal recognition particle receptor |

**Table S6** Properties of the MAGs reconstructed from the N2OR1 and N2OR2 metagenomes

| MAG ID | Completeness  (%) | Contamination  (%) | Genome  size (Mbp) | GC  (%) | Taxonomy | Closest species  (Genome accession number) | AAI  (%) |
| --- | --- | --- | --- | --- | --- | --- | --- |
| MH_R1_1 | 100 | 2.56 | 1.47 | 40.2 | phylum *Firmicutes* | *Thermosinus carboxydivorans* Nor1  (GCA 000169155) | 41.15 |
| MH_R1_12 | 85.62 | 3.47 | 0.85 | 32.9 | family *Comamonadaceae* | *Acidovorax caeni* (GCA 001298675) | 79.85 |
| MH_R1_16 | 96.63 | 0.54 | 0.97 | 37.4 | class *Bacteroidia* | *Lentimicrobium saccharophilum*  (GCA 001192835) | 51.19 |
| MH_R1_17 | 97.7 | 0 | 0.82 | 39.4 | class *Spirochaetia* | *Rectinema subterraneum* (GCA 009768935) | 53.82 |
| MH_R1_21 | 98.08 | 1.2 | 1.08 | 35.1 | family *Azonexaceae* | *Azonexus fungiphilus* (GCA 003634965) | 78.11 |
| MH_R1_24 | 96.97 | 2.04 | 1.43 | 33 | genus *Acidovorax* | *Acidovorax kalamii* (GCA 002245625) | 86.09 |
| MH_R1_2_6_11 | 95.07 | 0.47 | 0.61 | 33.5 | order *Burkholderiales* | *Hydromonas duriensis* (GCA 004363775) | 57.36 |
| MH_R1_5 | 97.81 | 3.58 | 0.76 | 36.4 | phylum *Firmicutes* | *Caldanaerobacter subterraneus* subsp. pacificus DSM 12653 (GCA 000156275) | 39.92 |
| MH_R1_8 | 93.88 | 0.91 | 0.91 | 34.5 | genus *Acinetobacter* | *Acinetobacter tjernbergiae* (GCA 000759995) | 88.57 |
| MS_R1_1 | 89.44 | 2.76 | 0.81 | 32 | order *Propionibacteriales* | *Propionicimonas paludicola* (GCA 002563675) | 63.94 |
| MS_R1_15 | 98.58 | 0.47 | 1.16 | 34.1 | genus *Oryzomicrobium* | *Oryzomicrobium terrae* (CP022579) | 89.88 |
| MS_R1_2 | 96.13 | 0.65 | 0.99 | 37.1 | family *Geobacteraceae* | *Geobacter thiogenes* (GCA 900167465) | 87.81 |
| MS_R1_22 | 100 | 0 | 1.09 | 34 | order *Micrococcales* | *Phycicoccus cremeus* (GCA 900111375) | 62.46 |
| MS_R1_8 | 84.09 | 6.85 | 1.26 | 36.5 | class *Anaerolineae* | *Levilinea saccharolytica* (GCA 001050255) | 52.57 |
| MH_R2_13 | 88.07 | 8.04 | 0.97 | 34.8 | order *Rhodocyclales* | *Azonexus fungiphilus* (GCA 003634965) | 67.72 |
| MH_R2_21 | 91.34 | 0.84 | 1.13 | 36.9 | class *Ignavibacteria* | *Ignavibacterium album* JCM 16511  (NC 017464) | 49.77 |
| MH_R2_22 | 87.59 | 4.99 | 0.51 | 33.1 | order *Burkholderiales* | *Hydromonas duriensis* (GCA 004363775) | 60.87 |
| MS_R2_10 | 95.4 | 0 | 0.84 | 39.4 | class *Spirochaetia* | *Rectinema subterraneum* (GCA 009768935) | 54.08 |
| MS_R2_11 | 99.37 | 0.16 | 1.16 | 33.3 | order *Rhizobiales* | *Siculibacillus lacustris* (GCA 004328075) | 64.62 |
| MS_R2_13 | 93.01 | 1.15 | 0.65 | 36.1 | class *Spirochaetia* | *Rectinema subterraneum* (GCA 009768935) | 55.48 |
| MS_R2_18 | 81.77 | 3.63 | 1.04 | 35.7 | order *Rhodocyclales* | *Zoogloea oryzae* (CP030061) | 65.68 |
| MS_R2_21 | 84.02 | 2.82 | 0.61 | 39 | order *Chlorobiales* | *Chlorobaculum limnaeum* (CP017305) | 65.7 |
| MS_R2_22 | 93.64 | 0.05 | 1.21 | 38.3 | class *Sphingobacteriia* | *Mucilaginibacter yixingensis*  (GCA 003050755) | 47.51 |
| MS_R2_24 | 86.78 | 1.92 | 0.79 | 36.1 | domain *Bacteria* | *Halomonas axialensis* (AP019517) | 38.82 |
| MS_R2_28 | 78.03 | 2.02 | 0.9 | 36.9 | order *Bacteroidales* | *Paludibacter propionicigenes* WB4 NC 014734 | 61.57 |
| MS_R2_3 | 90.59 | 1.4 | 0.75 | 33.3 | genus *Thiomonas* | *Thiomonas intermedia* CP020046 | 93.66 |
| MS_R2_30 | 89.47 | 0 | 0.98 | 36.6 | family *Holophagaceae* | *Geothrix fermentans* DSM 14018  (GCA 000428885) | 79.94 |
| MS_R2_31 | 84.19 | 3.95 | 0.98 | 36.3 | genus *Azovibrio* | *Azovibrio restrictus* DSM 23866  (GCA 000429665) | 98.33 |
| MS_R2_32 | 95.25 | 2.24 | 1.1 | 39.6 | order *Marinilabiliales* | *Prolixibacter bellariivorans* (GCA 009617915) | 56.23 |
| MS_R2_34 | 91.53 | 1.93 | 0.91 | 40.7 | phylum *Bacteroidetes* | Algibacter wandonensis (GCA 009807485) | 40.26 |

**Supplementary reference**

1. Löffler FE, Yan J, Ritalahti KM, Adrian L, Edwards EA, Konstantinidis KT, et al. *Dehalococcoides mccartyi* gen. nov., sp. nov., obligately organohalide-respiring anaerobic bacteria relevant to halogen cycling and bioremediation, belong to a novel bacterial class, *Dehalococcoidia* classis nov., order *Dehalococcoidales* ord. nov. and family *Dehalococcoidaceae* fam. nov., within the phylum *Chloroflexi*. Int J Syst Evol Microbiol. 2013;63:625-35.

2. Pruim R, Kaplan DT, Horton NJ. The mosaic package: helping students to think with data using R. R J. 2017;9:77.

3. Bolyen E, Rideout JR, Dillon MR, Bokulich NA, Abnet CC, Al-Ghalith GA, et al. Reproducible, interactive, scalable and extensible microbiome data science using QIIME 2. Nat Biotechnol. 2019;37:852-7.

4. Quast C, Pruesse E, Yilmaz P, Gerken J, Schweer T, Yarza P, et al. The SILVA ribosomal RNA gene database project: improved data processing and web-based tools. Nucleic Acids Res. 2012;41:D590-6.

5. Ritalahti KM, Amos BK, Sung Y, Wu Q, Koenigsberg SS, Löffler FE. Quantitative PCR targeting 16S rRNA and reductive dehalogenase genes simultaneously monitors multiple *Dehalococcoides* strains. Appl Environ Microbiol. 2006;72:2765-74.

6. Kim DD, Park D, Yoon H, Yun T, Song MJ, Yoon S. Quantification of *nosZ* genes and transcripts in activated sludge microbiomes with novel group-specific qPCR methods validated with metagenomic analyses. Water Res. 2020;185:116261.
